# Machine Learning-Based Integration Develops a Pyroptosis-Related lncRNA Model to Enhance the Predicted Value of Low-Grade Glioma Patients

**DOI:** 10.1155/2022/8164756

**Published:** 2022-05-19

**Authors:** Jie Wu, Lichun Lu, Chen Wang, Feng Jiang

**Affiliations:** ^1^Department of Neurosurgery, Suzhou Science & Technology Town Hospital, Gusu School, Nanjing Medical University, Suzhou 215153, China; ^2^Department of Neonatology, Obstetrics and Gynecology Hospital of Fudan University, Shanghai 200011, China

## Abstract

**Background:**

Molecular features have been included in the categorization of gliomas because they may be excellent predictors of tumor prognosis. Lower-grade glioma (LGGs, which comprise grade 2 and grade 3 gliomas) patients have a wide variety of outcomes. The goal of this research is to investigate a pyroptosis-based long noncoding RNA (lncRNA) profile and see whether it can be used to predict LGG prognosis.

**Methods:**

The Genotype-Tissue Expression (GTEx) and Cancer Genome Atlas (TCGA) datasets were utilized to get RNA data and clinical information for this research. Six considerably related lncRNAs (AL355574.1, AL355974.2, Z97989.1, SNAI3-AS1, LINC02593, and CYTOR) were selected using Cox regression (univariate and multivariate) and LASSO Cox regression. A variety of statistical techniques, including ROC curves, nomogram, and Kaplan-Meier curves, were utilized to verify the risk score's accuracy. Following that, bioinformatics studies were carried out to investigate the possible molecular processes that influence LGG prognosis. The variations in pathway enrichment were investigated using GSEA. The immune microenvironment inconsistencies were investigated using CIBERSORT, ESTIMATE, MCPcounter, TIMER algorithms, and ssGSEA.

**Results:**

We discovered six lncRNAs with distinct expression patterns that are linked to LGG prognosis. Kaplan-Meier studies showed a signature of high-risk lncRNAs associated with a poor prognosis for LGG. Furthermore, the AUC of the lncRNA signature was 0.763, indicating that they may be used to predict LGG prognosis. In predicting LGG prognosis, our risk assessment approach outperformed conventional clinicopathological characteristics. In the high-risk group of people, GSEA identified tumor-related pathways and immune-related pathways. Furthermore, T cell-related activities such as T cell coinhibition and costimulation, check point, APC coinhibition and costimulation, CCR, and inflammatory promoting were shown to be substantially different between the two groups in TCGA analysis. Immune checkpoints including PD-1, CTLA4, and PD-L1 were expressed differentially in the two groups as well.

**Conclusion:**

This study found that pyroptosis-based lncRNAs were useful in predicting LGG patients' survival, suggesting that they may be used as a therapeutic target in the future.

## 1. Introduction

Glioma is a cancerous tumor that poses a significant danger to human health throughout the globe. Complex histological kinds, significant variations in patient prognosis, and restricted therapeutic choices define it. The histopathology grade (World Health Organization) classifies glioma patients by convention in grades 1 to 4 and is widely used by doctors to evaluate prognosis and guided treatment [1]. The traditional TNM classification assessment findings, however, are not completely acceptable owing to the wide variety of patient survival within each grade. Scientists have found in recent years that IDH 1 and 2 mutations, as well as 1p/192 codeletion, play a major influence in glioma patient prognoses than TNM phases, thanks to extensive molecular biology study [2, 3]. The World Health Organization (WHO) made a significant modification in the categorization of gliomas in 2016, including molecular features in the classification [4]. Deep mining of gene expression data and the creation of more effective molecular markers have subsequently become research hotspots in the field of glioma.

Individuals between the ages of 20 and 40 are more likely to develop low-grade gliomas (LGGs, WHO category II and III gliomas) [5]. While LGGs are malignant and have a high mortality rate, their effect on society, families, and people is extraordinarily stressful, although LGG patients have a better prognosis compared to other CNS malignancies [6–8]. Treatment such as surgery, radiation, and chemotherapy have advanced in recent decades, and LGG patients' survival rate is very variable. Some patients have a one-year survival rate, while others have a 15-year survival rate. As a result, LGG is now confronted with two significant issues: the newly found molecular indicators (for example, IDH mutation and coding status 1p/19q) are unable to fully differentiate the prognosis of LGG, and the existing therapeutic approaches are unable to enhance the survival rate of patients [9]. Targeted treatment for LGG has recently been utilized in clinical trials; however, patient overall survival (OS) has not been as excellent as anticipated. As a result, it is critical to identify LGG's molecular subtypes and differentiate between high- and low-risk individuals, allowing for earlier diagnosis and better prognosis.

Noncoding RNAs (ncRNAs) having an intracellular length of more than 200 nucleotides are also known as long noncoding RNAs (lncRNAs) [10]. lncRNAs have no protein transcription feature yet play a vital role in a number of physiological activities, such as transcription regulation and the structure of nuclear domains. Meanwhile, lncRNAs are involved in a variety of cellular activities, including pyroptosis [11]. Results from these experiments show that lncRNAs are significantly upregulated in cancer cells and, as a result, may serve as useful diagnostic markers for LGG patients [12]. Furthermore, recent studies have shown a link between nonmutational gene expression regulation and treatment resistance, with lncRNAs acting as significant modulators of drug sensitivity to tumor cells [13, 14]. lncRNAs were believed to have a prognostic prediction potential and offer new treatment choices based on the very particular subtype of tumor cells.

Pyroptosis is a cell death that is coupled with the production and breakdown of proinflammatory mediators [15]. Pyroptosis-mediated inflammatory alterations promote carcinogenesis in normal cells and provide favorable tumor microenvironments for tumor growth [16, 17]. By stimulating the ERK 1/2 pathway, HMGB1 produced from pyroptotic epithelial cells promotes carcinogenesis of colitis-associated colorectal cancer [18]. Recent research found that pyroptosis induced in the tumor's core hypoxic area accelerated tumor growth and was linked to a worse survival rate [19]. Several research investigations have indicated that pyroptosis may be used as a biomarker to determine tumor prognosis [20, 21], and studies in the underlying mechanisms may provide new treatment options.

Several studies have shown that lncRNAs derived from pyroptosis have an effect on solid tumor cells (such as cervical cancer, digestive cancers, and breast cancer) [22–24]. The function of pyroptosis-based lncRNAs in LGG is unknown. In this research, we hypothesized that there are many lncRNAs associated with pyroptosis that may aid in forecasting the LGG prognosis. By integrating both the kinds and the molecular tumors, LGG heterogeneity may be more effectively shown, and a theoretical foundation for clinical diagnostics and prognosis can be provided. Finally, we created a novel pyroptosis score system based on six lncRNAs with the aim of accurately predicting patient prognosis.

## 2. Materials and Methods

### 2.1. Data Collection

The University of California Santa Cruz (UCSC) Xena website was used to acquire high-throughput RNA-seq data and clinical characteristics from TCGA database for 525 patients with LGG, as well as 1152 normal brain tissue samples from the GTEx project. FPKM normalized estimate and log2-based transformation were used to quantify the gene expression patterns. Then, from previous studies, we identified 33 pyroptosis-related genes, which are included in Table S1. Because TCGA cohort lacked normal brain tissue data, we used GTEx (Genotype-Tissue Expression) data to detect DEGs between normal and malignant tissues. The GTEx database is made up of over 7,000 autopsy samples from 449 living healthy human donors. Before comparing the two datasets, the expression data were standardized to FPKM values. The R software package “limma” was used to find DEGs under the absolute value of |log_2_FC| ≥ 1 and adj. *P* value < 0.05.

### 2.2. The Uncovering of Pyroptosis-Related lncRNAs and the Development of a Prognostic Model

To find pyroptosis-related lncRNAs, researchers used Pearson correlation analysis. The association between lncRNAs and pyroptosis genes was determined using their expression values. |R^2^| > 0.6 and *P* < 0.001 were our selection criteria. To create the pyroptosis-related lncRNA signature, we utilized LASSO Cox regression and univariate and multivariate Cox regression. Following that, we identified six pyroptosis-related lncRNAs as potential targets. Finally, using a prior method, the predictive model was built using six pyroptosis-related lncRNAs:

(1) $$\begin{array}{c} \text{risk score}=\overset{n}{\underset{i=1}{\sum }}\exp i*\beta i, \end{array}$$

where exp(*i*) and *β*(*i*) are the expression value of each lncRNAs linked to pyroptosis and the calculated regression coefficient in the formula.

### 2.3. Evaluating and Verifying the Adequacy of the Prognostic Signature

To discover the survival differences between the high- and low-risk groups, the Kaplan-Meier survival analysis was employed. In the current study, the gene expression and prognosis of different groups were examined using heat map and scatter plot. We evaluated the ROC curve's predictive abilities in order to determine the prediction's accuracy. Correlation analysis was used to establish relationships between the risk score and the patients' clinicopathological variables. To validate the independent prediction model, we have utilized univariate and multivariate Cox regression analyses.

### 2.4. Creating a Predicted Nomogram

A nomogram based on risk score and other clinicopathological characteristics was created to offer a reliable clinical prediction tool for LGG patients in terms of 1-, 3-, and 5-year survival. The calibration curves were then utilized for assessing the concordance between patients anticipated and observed.

### 2.5. Gene Set Enrichment Analysis

To identify differentially expressed functional phenotypes between the high-risk and low-risk groups, we utilized GSEA. The GSEA was used to categorize differentially expressed genes into two risk categories based on their expression profiles. The enriched gene sets were discovered using a *P* value < 0.05 and an FDR < 0.25. High- and low-risk groups were then analyzed using the Kyoto Encyclopedia of Genes and Genomes (KEGG) pathway method, which allowed a more detailed investigation of the pathways involved.

### 2.6. The Coexpression Network Created to Study lncRNA-mRNA Interactions

The links between pyroptosis-related lncRNAs and their target mRNAs were investigated using lncRNA-mRNA coexpression network constructed using Cytoscape, as well as the possible roles of the LGG six pyroptosis-related lncRNAs.

### 2.7. Analyses of Immunity and Gene Expression

Based on the lncRNA-related pyroptosis-related signature, the evaluation of the cellular components or cell-reacting immunological responses among high-risk and low-risk groups was conducted using CIBERSORT, ESTIMATE, MCP counter, and single-sample gene set enrichment analysis (ssGSEA). Using a heat map, different immune response differences were shown under various algorithms. The ssGSEA algorithm was used to conduct these additional comparisons and to evaluate the immunological activity of the tumor-infiltrating immune cell subgroups. We obtained immune checkpoint-related genes from the literature for analysis.

### 2.8. Statistical Analysis

For statistical analyses, R software version 4.0.2 and other R packages were utilized, with a 2-tailed *P* value < 0.05 signifying statistical significance. We used “survival” package to conduct univariate and multivariate Cox analysis. The “glmnet” package was used to conduct the LASSO Cox regression analysis, and 10 times cross-validation was utilized to find the optimum penalty parameter lambda. Kaplan-Meier analysis and survival curves were generated using “survival” package. The nomogram and calibration curve were created using the “rms” package. The “timeROC” package was used to analyze time-dependent ROC curves. Based on FDR, the Benjamini-Hochberg technique was utilized to determine the differentially expressed lncRNAs. The LGG DEGs were normalized using ssGSEA and compared to a genome using “GSVA” (R package).

## 3. Results

### 3.1. Prognosis of LGG Patient Tissue Samples with Pyroptosis-Associated lncRNAs

**Figure 1:**
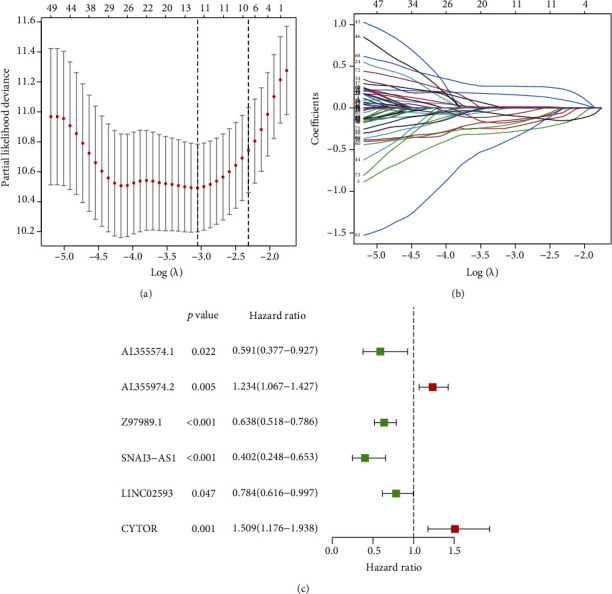
LASSO and Cox regression analysis were used to create a signature of pyroptosis-related lncRNAs. (a, b) The predictive pyroptosis-related lncRNA LASSO coefficient profiles. (c) Multivariate Cox regression analysis revealed that the forest plot of pyroptosis-associated lncRNAs is substantially linked to OS in LGG patients.

The following study comprised a total of 525 LGG patients. Table S2 contains the full clinical features of the patients. We identified 4 DEGs linked to pyroptosis (2 downregulated and 2 upregulated; Table S3). In addition, the Pearson correlation between the lncRNA and the associated genes was carried out for 859 pyroptosis-related lncRNAs with selection criterion of |R^2^| > 0.6 and *P* < 0.001 (Table S4). 77 lncRNAs were shown to be substantially associated with the survival time of LGG patients (*P* < 0.001; Table S5) in a univariate Cox regression analysis incorporating clinical survival data. For the predictive signature, LASSO regression and multivariate Cox regression filtered 6 lncRNAs (AL355574.1, AL355974.2, Z97989.1, SNAI3-AS1, LINC02593, and CYTOR) (Figure 1). AL355974.2 and CYTOR were the only lncRNAs with HR > 1, whereas AL355574.1, Z97989.1, SNAI3-AS1, and LINC02593 had HR < 1.

### 3.2. Validates the Predictive Signature of Six Pyroptosis-Related lncRNAs

**Figure 2:**
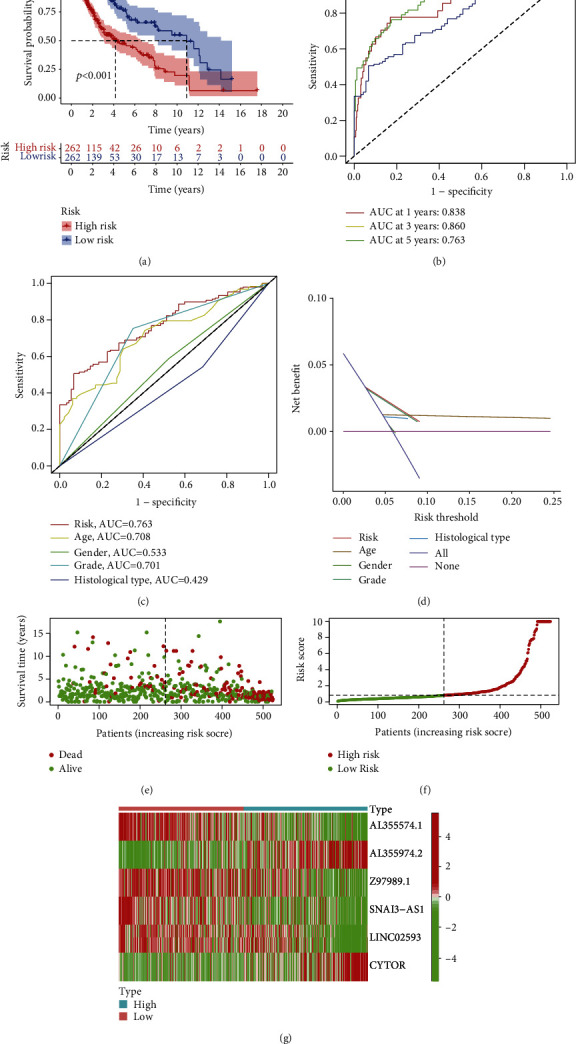
TCGA-based signature for pyroptosis-related lncRNAs. (a) The outcome of the Kaplan-Meier curves. (b) AUC values for predicting LGG survival rates at 1, 3, and 5 years. (c) The area under the curve (AUC) values of the risk variables. (d) The risk factors' DCA. (e) Plot of risk survival status. (f) The distribution of patient risk ratings. (g) Pyroptosis-related lncRNAs heat map in high- and low-risk groups.

Based on their respective median cut-off values, LGG patients were classified into two groups: high risk and low risk. The low-risk patients lived much longer and had a significantly better prognosis than the high-risk patients, according to a Kaplan-Meier survival curve study (Figure 2(a)). The ROC curve showed that using risk scores to predict LGG patient prognosis in 1-, 3-, and 5-years was reliable, with all AUC values greater than 0.7 (Figure 2(b)), and the signature had a 5-year AUC of 0.763, indicating that it outperformed conventional clinicopathological characteristics in predicting LGG patient prognosis. (Figures 2(c) and 2(d)). The risk ratings based on the prognostic signature of pyroptosis-related lncRNAs were subsequently utilized to assess LGG patients (Figure 2(e)). The survival rates of LGG patients were shown to be correlated with their risk score in a scatter dot plot; patients with a higher risk score had a shorter survival time. (Figure 2(f)). The heat map showed differential expression of lncRNAs associated with prognostic signatures in the low- and high-risk groups. Patients with increased risk had increased levels of risk factors (AL355974.2, CYTOR), while patients at reduced risk had increased levels of protective factors (AL355574.1, Z97989.1, SNAI3-AS1, and LINC02593) (Figure 2(g)).

### 3.3. Assess the Ability to Predict lncRNA Risk Signature Linked to Pyroptosis

**Figure 3:**
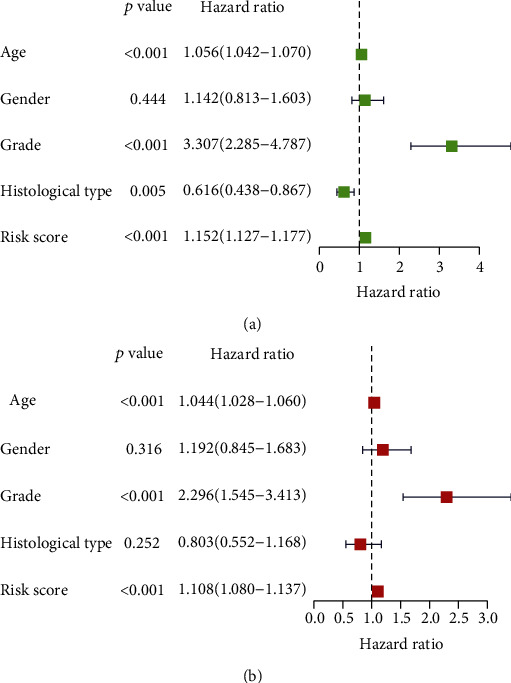
Cox analysis for pyroptosis-related lncRNAs, both univariate and multivariate. (a) Univariate analysis. (b) Multivariate analysis.

**Figure 4:**
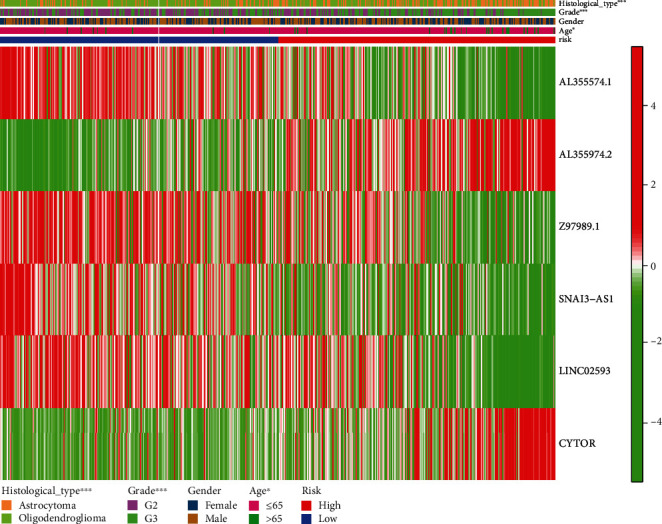
Prognostic signature and clinicopathological manifestations of pyroptosis-related lncRNAs.

Next, we used a cox regression analysis to investigate if the pyroptosis-related lncRNA prediction signature in LGG patients was an independent prognostic factor. With the exception of gender (*P* = 0.444), age (*P* < 0.001), grade (*P* < 0.001), histological type (*P* = 0.005), and pyroptosis-related lncRNA prediction risk score (*P* < 0.001) were all significantly linked with survival time in univariate analysis (Figure 3(a)). Age (*P* < 0.001), grade (*P* < 0.001), and the predictive risk score for pyroptosis-related lncRNAs (*P* < 0.001) were all shown to be significantly linked with survival time in multivariate analysis (Figure 3(b)). The heat map for the predictive signature of pyroptosis-related lncRNAs and clinicopathological symptoms was also examined (Figure 4). All these data indicate that the risk score associated with pyroptosis lncRNAs independently predicts prognosis in LGG patients.

### 3.4. Quantification of Clinical Indicators Using Nomograms and Evaluation of the Predictive Accuracy of Risk Scores

**Figure 5:**
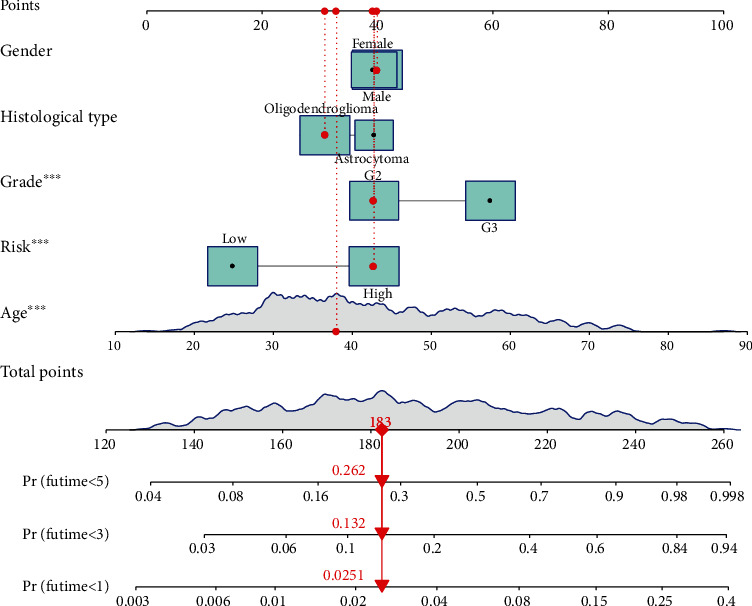
A nomogram for both clinicopathological and prognostic lncRNAs associated with pyroptosis.

**Figure 6:**
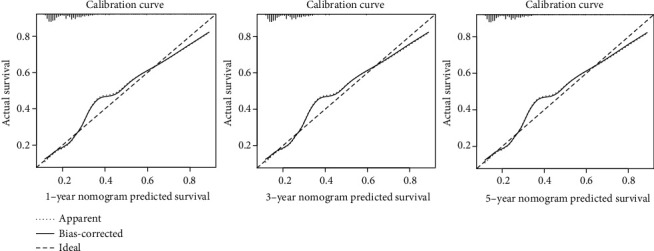
Calibration curve for the predicted survival nomograms for 1, 3, and 5 years.

The nomogram based on clinicopathological characteristics and the prognostic signature of pyroptosis-related lncRNAs was used to generate the score for assessing the precision of the model in this section. To correctly predict the 1-, 3-, and 5-year survival time in LGG patients, we created a nomogram (Figure 5) that included various clinicopathological parameters such as age, gender, histological type, grade, and pyroptosis-related lncRNAs risk score. The calibration curve study revealed the agreement between LGG patients' anticipated and observed 1-, 3-, and 5-year OS (Figure 6).

### 3.5. Building and Analyzing a lncRNA-mRNA Expression Network

**Figure 7:**
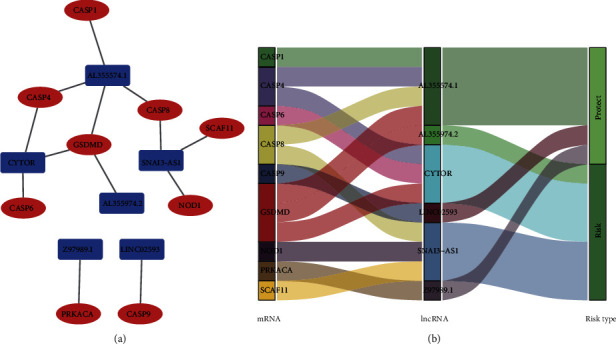
(a) The novel lncRNA's connection to mRNA expression. (b) Sankey diagram of the LGG lncRNA network.

**Figure 8:**
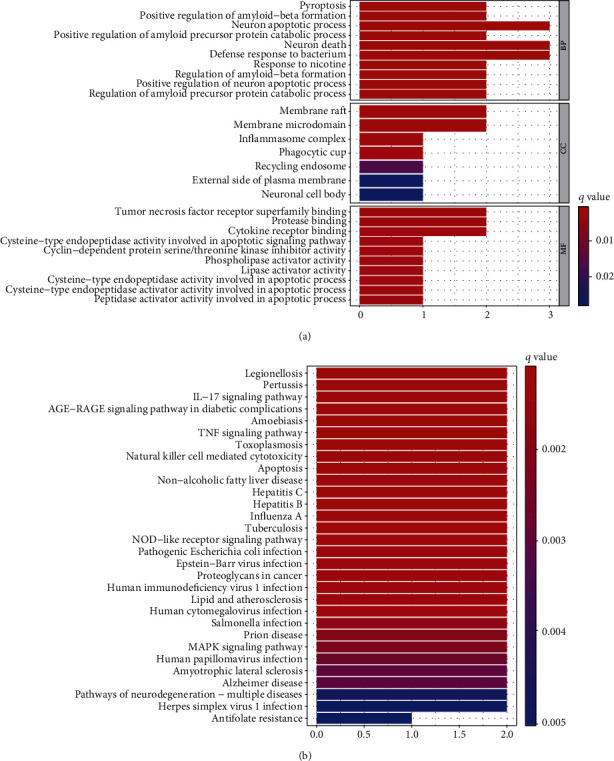
GO and KEGG analysis of genes linked to pyroptosis. (a) GO and (b) KEGG.

To investigate the possible roles of the six pyroptosis-related lncRNAs in LGG, we built a lncRNA-mRNA coexpression network in Cytoscape. We found 15 connections between six lncRNAs and nine associated mRNAs (Figure 7(a)). The Sankey diagram showed the connection between the nine mRNAs and the six long noncoding RNAs (risk/protective) (Figure 7(b)). Six lncRNAs were shown to have a substantial correlation with the prognostic signature's nine mRNAs. Meanwhile, BP was shown to be involved in pyroptosis, execution phase of apoptosis, and positive regulation of IL-1*β* production according to GO and KEGG analyses. CC was involved in inflammasome complex, cAMP-dependent protein kinase complex, and membrane raft. KEGG was enriched in the NOD-like receptor signaling pathway, apoptosis, and lipid and atherosclerosis (Figure 8).

### 3.6. Gene Set Enrichment Analyses

**Figure 9:**
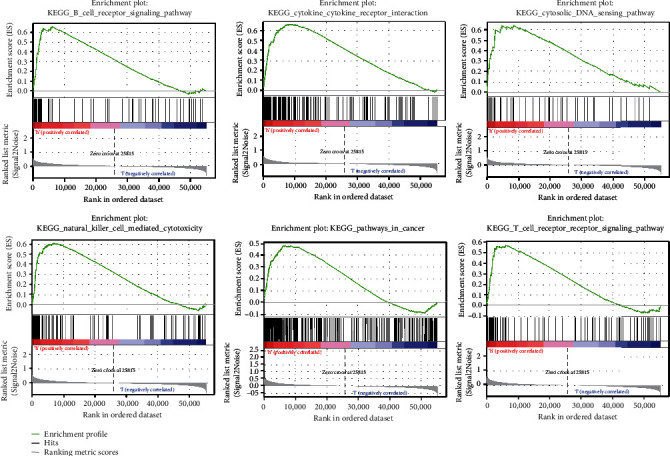
TCGA-based GSEA of pyroptosis-related lncRNAs.

In order to show the possible route and functions of the pyroptosis-related signature of the LGG, we used GSEA to compare the high-risk and low-risk groups. The results showed that the pyroptosis-related signature lncRNAs in the high-risk group were significantly enriched by a signaling pathway of a B cell receptor, cytokine receptor, a cytosolic DNA sensing path, natural killer cell-mediated cytotoxicity, a cancer pathway, and a signal pathway of a T cell receptor (Figure 9). Our findings will help researchers discover novel personalized therapies and execute full-process management of LGG patients with different risk categories in the future.

### 3.7. Gene Expression and Immunity

**Figure 10:**
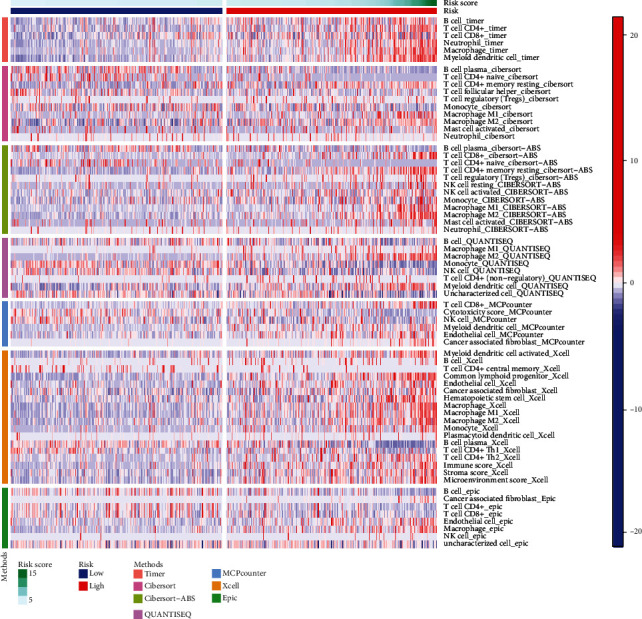
A heat map of immunological responses between high- and low-risk groups using the CIBERSORT, ESTIMATE, MCP counter, ssGSEA, and TIMER algorithms.

**Figure 11:**
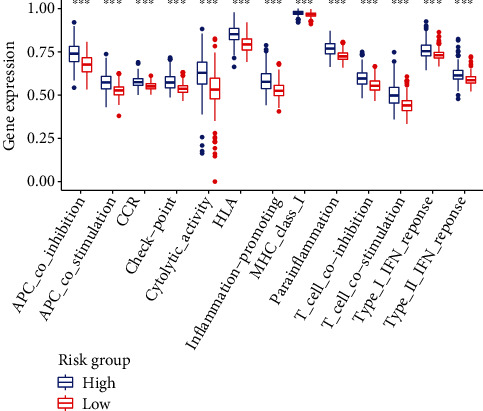
Immune cell subpopulations and associated roles in ssGSEA.

**Figure 12:**
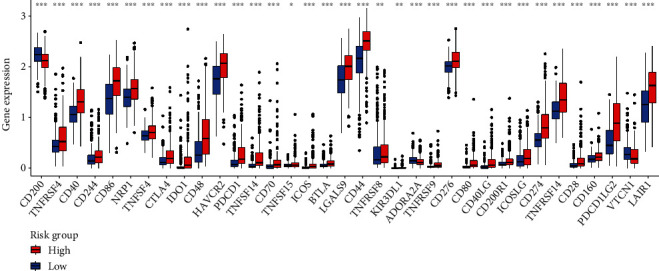
Immune checkpoint expression in high- and low-risk populations.

Figure 10 displays the heat map on the basis of CIBERSORT, ESTIMATE, the MCP counter, enrichment analyses (ssGSEA), and TIMER of the immunological reactions. The ssGSEA of TCGA-KIRC data revealed that APC coinhibition and costimulation, CCR, check-point, cytolytic activity, HLA, inflammation-promoting, MHC class I, parainflammation, T cell coinhibition and costimulation, and type I and II IFN response were significantly different between the risk group immune cell subpopulations (Figure 11). Because checkpoint inhibitor-based immunotherapies are so important, we looked into the differences in immune checkpoint expression between the two groups further. Most immunological checkpoints, such as PD-L1, PD-1, and CTLA4, showed a significant variation in expression (Figure 12).

## 4. Discussion

Low-grade gliomas are the most frequent primary tumors in the central nervous system. They are physiologically and clinically quite diverse. The standard LGG treatment is currently postoperative chemoradiotherapy with maximal surgical resection. LGG, on the other hand, often develops resistance to treatment and evolves to high-grade aggressive glioma [25, 26]. Novel variables influencing LGG prognosis are therefore urgently required.

Previous research has shown that lncRNAs, a key noncoding RNA family member, play a role in the invasion and development of LGG. Glioma cell growth and metastasis are impeded in vitro when the lncRNA PTENP1 is overexpressed [27]; according to the study by Wang et al., PDIA3P1, a hypoxia-induced long noncoding RNA, promotes mesenchymal transition in glioblastoma via sponging miR-124-3p [28]. He et al. discovered that the lncRNA DCGR5 suppresses tumor growth in glioma cells through the miR-21/Smad7 and miR-23a/PTEN axis [29]. There are also some previous LGG-related prognostic models with good predictive efficacy [30–33]. Pyroptosis is thought to be linked to the proliferation and migration of cancer cells. Pyroptosis increases cancer cell inflammatory cell death and inhibits cancer cell growth and migration. In cancer cells, the expression of certain pyroptotic inflammasomes has been shown to decrease. Recent research has focused on chemicals that influence pyroptotic inflammasomes and promote pyroptosis. These molecules include noncoding RNAs and other types of molecules that may be used as targets for successful cancer therapy in the future. Pyroptosis also releases inflammatory chemicals, which suppress tumor growth. It may, however, impair the body's immunological response to tumor cells and promote tumor development in certain malignancies [34–37].

The prior research used a variety of survival and prognostic analytic techniques. “Gene mutations and copy number variations analyses,” according to some research, may be utilized to determine the difference between high- and low-risk populations [38]. We calculated the risk score in this study using six previously unreported pyroptosis-related lncRNAs (AL355574.1, AL355974.2, Z97989.1, SNAI3-AS1, LINC02593, and CYTOR), then confirmed its independent predictive ability using multivariate regression analysis with other clinically relevant parameters and the accuracy using a receiver operating characteristic curve. Finally, the nomogram based on additional clinical factors demonstrated the benefit of the prediction score of pyroptosis-related lncRNAs. In high-risk patients, GSEA revealed an enrichment of the cytosolic DNA sensing route, focal adhesion, natural killer cell-mediated cytotoxicity, B cell receptor signaling pathway, cancer pathways, and apoptosis. Cytosolic DNA sensors are virtually ubiquitous, contrasting with the TLR9, a endosomal DNA sensor, expressed from the immune system. In glioma, integrin binding and growth factor receptor signaling can activate focal adhesion, leading to cell cycle progression and cell invasion [39]. It is widely assumed that lncRNAs do not directly encode proteins but rather influence gene expression via a number of mechanisms to promote carcinogenesis and tumor metastasis [40]. The biological function and signal route of the lncRNA-mRNA regulatory network were investigated in this research. Numerous signaling pathways were involved in the regulation of lncRNA-mRNA, with the usual trimolecular regulatory network (lncRNA-miRNA-TF/gene) being well-characterized in other malignant tumors [41, 42]. lncRNA influenced the function of downstream target genes and slowed illness progression by sharing similar miRNA-binding sites [43]. In hepatocellular cancer, Li et al. discovered that the long noncoding RNA SNAI3-AS1 enhances proliferation and metastasis mediated by PEG10 by decoying miR-27a-3p and miR-34a-5p [44]. By interacting with NCL and Sam68, the lncRNA CYTOR promotes colorectal cancer development [45]. Our research identified a novel target for tumor progression studies.

Xu et al. showed that under hypoxia, nuclear PD-L1 induces pyroptosis in cancer cells through GSDMC; more importantly, they discovered that this leads to tumor necrosis, which is a marker of poor prognosis in solid tumors. Only a few research have looked at the connection between ICI and pyroptosis. Increasing data indicate that miRNA and lncRNA play a key role in pyroptosis control. TLR4 stimulates the PI3K/AKT pathway through lncRNA-F630028010Rik to increase microglial pyroptosis after spinal cord injury [46]. Surprisingly, lncRNA has a role in pyroptosis control.

Pyroptosis is a novel kind of cell death that has the potential to revolutionize tumor therapy. Many important questions, such as the relationship between pyroptosis and other cell deaths, as well as host immunogenicity, remain unanswered. As a result, this research looked at LGG pyroptosis, which may help with therapeutic options. There are some limitations in this study; firstly, we have based our bioinformatics analysis entirely on public databases which have not been validated experimentally or with clinical samples. Our results should also be used with care due to the limited clinical evidence. The prognostic prediction model established in this research, in general, requires further validation.

## 5. Conclusion

The prognosis of LGG may be predicted by certain pyroptosis-associated lncRNAs.

## Data Availability

The datasets analyzed in this study are available in The Cancer Genome Atlas (TCGA).

## Supplementary Materials

Supplementary Materials Supplementary File Table S1: 33 pyroptosis-related genes from prior reviews. Supplementary File Table S2: patients' clinical characteristics from TCGA-LGG. Supplementary File Table S3: 4 pyroptosis-related DEGs from TCGA-LGG. Supplementary File Table S4: 859 pyroptosis-related lncRNAs. Supplementary File Table S5: 77 significant pyroptosis-related lncRNAs after univariate Cox analysis.
