# Supplementary material for: Machine Learning-Based Integration Develops a Pyroptosis-Related lncRNA Model to Enhance the Predicted Value of Low-Grade Glioma Patients
Source: J Oncol. 2022 May 19;2022:8164756. doi: 10.1155/2022/8164756 (PMC9135526; doi:10.1155/2022/8164756)
Supplement: Supplementary Materials — Supplementary File Table S1: 33 pyroptosis-related genes from prior reviews. Supplementary File Table S2: patients' clinical characteristics from TCGA-LGG. Supplementary File Table S3: 4 pyroptosis-related DEGs from TCGA-LGG. Supplementary File Table S4: 859 pyroptosis-related lncRNAs. Supplementary File Table S5: 77 significant pyroptosis-related lncRNAs after univariate Cox analysis. [file 8164756.f1.zip › Table S2.docx]

Table S2. Patients' clinical characteristics from TCGA-LGG.

| Clinical characteristic |  | TCGA-LGG |
| --- | --- | --- |
| Age | <60 | 455 |
|  | ≥60 | 70 |
|  | unknown | 0 |
| Gender | Female | 238 |
|  | Male | 287 |
| Race | White | 484 |
|  | Other | 31 |
| Grade | II | 258 |
|  | III | 266 |
|  | unknown | 1 |
| Radiation therapy | No | 174 |
|  | Yes | 284 |
|  | unknown | 67 |
| IDH mutation | No | 34 |
|  | Yes | 91 |
|  | unknown | 400 |
| Motor change | No | 355 |
|  | Yes | 122 |
|  | unknown | 48 |
| Sensor change | No | 392 |
|  | Yes | 72 |
|  | unknown | 61 |
| Seizure history | No | 183 |
|  | Yes | 309 |
|  | unknown | 33 |
| Headache history | No | 301 |
|  | Yes | 175 |
|  | unknown | 49 |
