# Supplementary material for: Machine Learning-Based Integration Develops a Pyroptosis-Related lncRNA Model to Enhance the Predicted Value of Low-Grade Glioma Patients
Source: J Oncol. 2022 May 19;2022:8164756. doi: 10.1155/2022/8164756 (PMC9135526; doi:10.1155/2022/8164756)
Supplement: Supplementary Materials — Supplementary File Table S1: 33 pyroptosis-related genes from prior reviews. Supplementary File Table S2: patients' clinical characteristics from TCGA-LGG. Supplementary File Table S3: 4 pyroptosis-related DEGs from TCGA-LGG. Supplementary File Table S4: 859 pyroptosis-related lncRNAs. Supplementary File Table S5: 77 significant pyroptosis-related lncRNAs after univariate Cox analysis. [file 8164756.f1.zip › Table S3.docx]

**Table S3.** 4 pyroptosis-related DEGs from TCGA-LGG

| gene | conMean | treatMean | logFC | pValue | fdr |
| --- | --- | --- | --- | --- | --- |
| CASP3 | 2.644553 | 3.760722 | 1.116169 | 2.91E-193 | 3.63E-192 |
| GSDMB | 3.148704 | 1.348007 | -1.8007 | 1.95E-208 | 4.87E-207 |
| NLRP1 | 6.072677 | 4.815746 | -1.25693 | 9.44E-128 | 4.72E-127 |
| TNF | 0.129873 | 1.363849 | 1.233977 | 5.00E-172 | 4.17E-171 |
