# Supplementary material for: Machine Learning-Based Integration Develops a Pyroptosis-Related lncRNA Model to Enhance the Predicted Value of Low-Grade Glioma Patients
Source: J Oncol. 2022 May 19;2022:8164756. doi: 10.1155/2022/8164756 (PMC9135526; doi:10.1155/2022/8164756)
Supplement: Supplementary Materials — Supplementary File Table S1: 33 pyroptosis-related genes from prior reviews. Supplementary File Table S2: patients' clinical characteristics from TCGA-LGG. Supplementary File Table S3: 4 pyroptosis-related DEGs from TCGA-LGG. Supplementary File Table S4: 859 pyroptosis-related lncRNAs. Supplementary File Table S5: 77 significant pyroptosis-related lncRNAs after univariate Cox analysis. [file 8164756.f1.zip › Table S4.docx]

**Table S4**. 859 pyroptosis-related lncRNAs.

| ferrGene | lncRNA | cor | pvalue | Regulation |
| --- | --- | --- | --- | --- |
| NLRP2 | AC090241.2 | 0.515674 | 2.82E-37 | postive |
| PRKACA | AC090241.2 | 0.502741 | 3.13E-35 | postive |
| CASP8 | AL731569.1 | -0.50585 | 1.03E-35 | negative |
| GSDMB | AL731569.1 | 0.568561 | 1.26E-46 | postive |
| NLRP2 | AC015468.3 | 0.531454 | 6.78E-40 | postive |
| GSDMD | AL158055.1 | -0.52347 | 1.49E-38 | negative |
| TIRAP | AL158055.1 | 0.519927 | 5.72E-38 | postive |
| GSDMD | AC022150.3 | -0.50827 | 4.28E-36 | negative |
| CASP1 | AC023762.1 | -0.50777 | 5.13E-36 | negative |
| CASP8 | AC023762.1 | -0.50412 | 1.91E-35 | negative |
| NOD1 | AC023762.1 | -0.52954 | 1.43E-39 | negative |
| NLRP2 | AL021395.1 | 0.56873 | 1.17E-46 | postive |
| GSDMB | AC009065.8 | 0.607541 | 1.11E-54 | postive |
| CASP6 | C2orf27A | -0.53309 | 3.56E-40 | negative |
| CASP8 | C2orf27A | -0.5411 | 1.45E-41 | negative |
| GSDMD | C2orf27A | -0.56967 | 7.68E-47 | negative |
| PRKACA | C2orf27A | -0.56291 | 1.52E-45 | negative |
| PJVK | AC087742.1 | 0.630456 | 5.80E-60 | postive |
| NLRP2 | DLGAP1-AS4 | 0.550882 | 2.59E-43 | postive |
| CASP1 | LINC01771 | -0.52053 | 4.56E-38 | negative |
| PYCARD | LINC01771 | -0.50593 | 9.98E-36 | negative |
| CASP6 | AC004233.3 | -0.51748 | 1.43E-37 | negative |
| CASP8 | AC004233.3 | -0.57495 | 7.14E-48 | negative |
| PYCARD | MRPL20-AS1 | 0.543111 | 6.41E-42 | postive |
| CASP1 | LINC02440 | -0.53558 | 1.33E-40 | negative |
| CASP4 | LINC02440 | -0.52116 | 3.59E-38 | negative |
| CASP6 | LINC02440 | -0.58489 | 7.22E-50 | negative |
| CASP8 | LINC02440 | -0.62705 | 3.76E-59 | negative |
| NOD1 | LINC02440 | -0.60539 | 3.29E-54 | negative |
| CASP1 | AC139768.1 | -0.57659 | 3.38E-48 | negative |
| CASP3 | AC139768.1 | -0.53073 | 9.01E-40 | negative |
| CASP4 | AC139768.1 | -0.62999 | 7.52E-60 | negative |
| CASP6 | AC139768.1 | -0.66352 | 2.06E-68 | negative |
| CASP8 | AC139768.1 | -0.64879 | 1.62E-64 | negative |
| NOD1 | AC139768.1 | -0.56372 | 1.06E-45 | negative |
| GSDMB | AC091946.2 | 0.596302 | 3.02E-52 | postive |
| SCAF11 | AL513477.2 | -0.5066 | 7.83E-36 | negative |
| GSDMB | AL136531.1 | 0.546362 | 1.69E-42 | postive |
| GSDMB | AC073592.1 | 0.510533 | 1.88E-36 | postive |
| NOD1 | PINK1-AS | 0.513476 | 6.36E-37 | postive |
| NLRP2 | LINC02458 | 0.557069 | 1.89E-44 | postive |
| GPX4 | AC008608.2 | 0.523206 | 1.65E-38 | postive |
| PRKACA | DOCK9-DT | 0.511507 | 1.31E-36 | postive |
| PYCARD | AC099568.1 | 0.500804 | 6.23E-35 | postive |
| CASP6 | AC124312.2 | -0.54068 | 1.72E-41 | negative |
| GSDMD | AC124312.2 | -0.62681 | 4.30E-59 | negative |
| PYCARD | AC124312.2 | -0.53967 | 2.59E-41 | negative |
| CASP8 | LINC01918 | -0.50649 | 8.16E-36 | negative |
| CASP6 | AP000766.1 | -0.51534 | 3.19E-37 | negative |
| GSDMD | AP000766.1 | -0.54971 | 4.22E-43 | negative |
| CASP8 | FAM181A-AS1 | 0.535697 | 1.27E-40 | postive |
| GSDMB | AL139123.1 | 0.501907 | 4.21E-35 | postive |
| NLRP2 | AC015712.7 | 0.574277 | 9.68E-48 | postive |
| GSDMB | AC074117.1 | 0.632119 | 2.31E-60 | postive |
| GSDMB | AC040162.3 | 0.766197 | ###### | postive |
| CASP1 | USP30-AS1 | 0.664294 | 1.26E-68 | postive |
| CASP4 | USP30-AS1 | 0.715797 | 3.15E-84 | postive |
| CASP8 | USP30-AS1 | 0.58719 | 2.43E-50 | postive |
| GSDMD | USP30-AS1 | 0.564433 | 7.79E-46 | postive |
| NLRC4 | USP30-AS1 | 0.566883 | 2.65E-46 | postive |
| PYCARD | USP30-AS1 | 0.505396 | 1.21E-35 | postive |
| GSDMB | LINC02293 | 0.543926 | 4.59E-42 | postive |
| GSDMB | Z84485.1 | 0.505629 | 1.11E-35 | postive |
| PJVK | Z84485.1 | 0.629867 | 8.03E-60 | postive |
| SCAF11 | AC098484.2 | -0.53596 | 1.14E-40 | negative |
| TIRAP | AP003392.4 | 0.519225 | 7.45E-38 | postive |
| CASP1 | AL355574.1 | -0.52549 | 6.87E-39 | negative |
| CASP4 | AL355574.1 | -0.55732 | 1.70E-44 | negative |
| CASP8 | AL355574.1 | -0.52613 | 5.36E-39 | negative |
| GSDMD | AL355574.1 | -0.55435 | 6.00E-44 | negative |
| GSDMB | AC008764.6 | 0.563303 | 1.28E-45 | postive |
| GSDMB | AL450384.2 | 0.730345 | 2.77E-89 | postive |
| GSDMB | AL021707.4 | 0.562473 | 1.83E-45 | postive |
| PYCARD | LINC02019 | 0.533595 | 2.92E-40 | postive |
| CASP8 | DRAIC | -0.51681 | 1.84E-37 | negative |
| NLRP2 | ATP2B1-AS1 | 0.531069 | 7.89E-40 | postive |
| IL18 | AF127936.1 | 0.510457 | 1.93E-36 | postive |
| NLRC4 | AF127936.1 | 0.586622 | 3.19E-50 | postive |
| NLRP3 | AF127936.1 | 0.597736 | 1.50E-52 | postive |
| CASP4 | AP002807.1 | 0.502221 | 3.77E-35 | postive |
| GSDMD | AP002807.1 | 0.592715 | 1.73E-51 | postive |
| GSDMB | AL592211.1 | 0.556733 | 2.18E-44 | postive |
| PJVK | AL592211.1 | 0.50432 | 1.78E-35 | postive |
| CASP1 | AC093627.7 | 0.630254 | 6.49E-60 | postive |
| IL18 | AC093627.7 | 0.706564 | 3.51E-81 | postive |
| NLRC4 | AC093627.7 | 0.699909 | 4.66E-79 | postive |
| NLRP3 | AC093627.7 | 0.648677 | 1.72E-64 | postive |
| PYCARD | AC093627.7 | 0.716805 | 1.44E-84 | postive |
| GSDMB | AC008764.8 | 0.587356 | 2.25E-50 | postive |
| GSDMB | IL21R-AS1 | 0.540182 | 2.10E-41 | postive |
| CASP8 | LINC00886 | 0.580897 | 4.65E-49 | postive |
| GSDMD | LINC00886 | 0.530251 | 1.09E-39 | postive |
| GSDMB | AP002812.5 | 0.543591 | 5.27E-42 | postive |
| GSDMB | AC008875.1 | 0.530481 | 9.93E-40 | postive |
| GSDMB | AP000708.1 | -0.55667 | 2.24E-44 | negative |
| CASP1 | AL049749.1 | -0.59974 | 5.56E-53 | negative |
| CASP4 | AL049749.1 | -0.54101 | 1.50E-41 | negative |
| CASP6 | AL049749.1 | -0.58327 | 1.54E-49 | negative |
| CASP8 | AL049749.1 | -0.62316 | 3.11E-58 | negative |
| IL18 | AL049749.1 | -0.51583 | 2.66E-37 | negative |
| NOD1 | AL049749.1 | -0.61041 | 2.55E-55 | negative |
| GSDMD | LINC01273 | 0.515299 | 3.24E-37 | postive |
| CASP1 | AL136964.1 | -0.61603 | 1.37E-56 | negative |
| CASP4 | AL136964.1 | -0.54564 | 2.27E-42 | negative |
| CASP6 | AL136964.1 | -0.56966 | 7.73E-47 | negative |
| CASP8 | AL136964.1 | -0.53292 | 3.82E-40 | negative |
| GSDMD | AL136964.1 | -0.51549 | 3.01E-37 | negative |
| IL18 | AL136964.1 | -0.58846 | 1.33E-50 | negative |
| NLRC4 | AL136964.1 | -0.50463 | 1.60E-35 | negative |
| NOD1 | AL136964.1 | -0.59864 | 9.60E-53 | negative |
| PYCARD | AL136964.1 | -0.60345 | 8.77E-54 | negative |
| GSDMD | AL355974.2 | 0.501812 | 4.36E-35 | postive |
| TIRAP | AC008840.1 | 0.536584 | 8.91E-41 | postive |
| CASP1 | LINC02381 | 0.603612 | 8.07E-54 | postive |
| CASP4 | LINC02381 | 0.507155 | 6.41E-36 | postive |
| CASP6 | LINC02381 | 0.514869 | 3.80E-37 | postive |
| IL18 | LINC02381 | 0.579645 | 8.31E-49 | postive |
| NLRC4 | LINC02381 | 0.519272 | 7.32E-38 | postive |
| PYCARD | LINC02381 | 0.586538 | 3.31E-50 | postive |
| GSDMB | AC106886.3 | 0.578426 | 1.46E-48 | postive |
| PYCARD | HOMER3-AS1 | 0.524749 | 9.14E-39 | postive |
| PJVK | AC132872.2 | 0.516414 | 2.14E-37 | postive |
| CASP1 | HOTAIRM1 | 0.504493 | 1.67E-35 | postive |
| CASP3 | HOTAIRM1 | 0.507527 | 5.61E-36 | postive |
| CASP4 | HOTAIRM1 | 0.568586 | 1.25E-46 | postive |
| CASP6 | HOTAIRM1 | 0.65202 | 2.36E-65 | postive |
| CASP8 | HOTAIRM1 | 0.565221 | 5.51E-46 | postive |
| GSDMD | HOTAIRM1 | 0.623448 | 2.66E-58 | postive |
| CASP8 | AC073389.3 | -0.51108 | 1.53E-36 | negative |
| CASP1 | LINC02611 | 0.829778 | ###### | postive |
| CASP4 | LINC02611 | 0.730094 | 3.41E-89 | postive |
| CASP6 | LINC02611 | 0.529301 | 1.57E-39 | postive |
| CASP8 | LINC02611 | 0.667292 | 1.90E-69 | postive |
| GSDMD | LINC02611 | 0.595129 | 5.36E-52 | postive |
| IL18 | LINC02611 | 0.78349 | ###### | postive |
| NLRC4 | LINC02611 | 0.808653 | ###### | postive |
| NLRP3 | LINC02611 | 0.622065 | 5.60E-58 | postive |
| NOD1 | LINC02611 | 0.592101 | 2.33E-51 | postive |
| PYCARD | LINC02611 | 0.803377 | ###### | postive |
| PJVK | NALT1 | 0.558321 | 1.10E-44 | postive |
| CASP9 | AC068057.1 | -0.52766 | 2.98E-39 | negative |
| CASP1 | LINC00632 | -0.54522 | 2.71E-42 | negative |
| CASP4 | LINC00632 | -0.58808 | 1.60E-50 | negative |
| CASP6 | LINC00632 | -0.56335 | 1.25E-45 | negative |
| CASP8 | LINC00632 | -0.56638 | 3.31E-46 | negative |
| GSDMD | LINC00632 | -0.57458 | 8.46E-48 | negative |
| CASP8 | AC069224.1 | -0.5091 | 3.16E-36 | negative |
| PRKACA | AC069224.1 | -0.50864 | 3.75E-36 | negative |
| GSDMB | SLC25A25-AS1 | 0.771946 | ###### | postive |
| GSDMB | AL021368.1 | 0.505186 | 1.30E-35 | postive |
| PLCG1 | AC018766.1 | 0.517935 | 1.21E-37 | postive |
| GSDMD | RGMB-AS1 | -0.50467 | 1.57E-35 | negative |
| CASP1 | AL022313.4 | -0.66918 | 5.69E-70 | negative |
| CASP4 | AL022313.4 | -0.62565 | 8.08E-59 | negative |
| CASP6 | AL022313.4 | -0.65964 | 2.29E-67 | negative |
| CASP8 | AL022313.4 | -0.6652 | 7.13E-69 | negative |
| GSDMD | AL022313.4 | -0.55622 | 2.71E-44 | negative |
| IL18 | AL022313.4 | -0.57899 | 1.13E-48 | negative |
| NLRC4 | AL022313.4 | -0.5354 | 1.43E-40 | negative |
| NOD1 | AL022313.4 | -0.68461 | 2.15E-74 | negative |
| PYCARD | AL022313.4 | -0.54327 | 6.01E-42 | negative |
| GSDMB | AL121890.2 | 0.618238 | 4.30E-57 | postive |
| PJVK | AL121890.2 | 0.511468 | 1.33E-36 | postive |
| PRKACA | TRAF3IP2-AS1 | -0.55915 | 7.73E-45 | negative |
| CASP4 | AC092368.3 | 0.512107 | 1.05E-36 | postive |
| SCAF11 | CELF2-AS1 | 0.552041 | 1.59E-43 | postive |
| GSDMB | MHENCR | 0.549977 | 3.77E-43 | postive |
| PJVK | MHENCR | 0.573713 | 1.25E-47 | postive |
| NLRP2 | KRTAP5-AS1 | 0.509426 | 2.81E-36 | postive |
| CASP1 | AC015961.1 | -0.5476 | 1.01E-42 | negative |
| CASP4 | AC015961.1 | -0.50097 | 5.88E-35 | negative |
| IL18 | AC015961.1 | -0.50031 | 7.43E-35 | negative |
| NOD1 | AC015961.1 | -0.53528 | 1.50E-40 | negative |
| PYCARD | AC015961.1 | -0.5137 | 5.86E-37 | negative |
| NLRP2 | LINC01361 | 0.550867 | 2.60E-43 | postive |
| GPX4 | AC006942.1 | 0.517731 | 1.31E-37 | postive |
| GSDMB | AC006942.1 | 0.561424 | 2.89E-45 | postive |
| CASP8 | AL008718.2 | -0.52962 | 1.39E-39 | negative |
| GSDMB | AC084824.5 | 0.627252 | 3.38E-59 | postive |
| PJVK | AC084824.5 | 0.526145 | 5.34E-39 | postive |
| CASP4 | AL391807.1 | -0.5413 | 1.34E-41 | negative |
| CASP8 | AL391807.1 | -0.50488 | 1.46E-35 | negative |
| GSDMB | AL391807.1 | 0.572612 | 2.06E-47 | postive |
| PJVK | AL391807.1 | 0.573308 | 1.50E-47 | postive |
| GSDMB | AL117336.2 | 0.640758 | 1.74E-62 | postive |
| GSDMB | MUC20-OT1 | 0.526918 | 3.96E-39 | postive |
| GSDMB | AC007608.3 | 0.527983 | 2.63E-39 | postive |
| GSDMB | RFPL3S | 0.766353 | ###### | postive |
| CASP1 | LINC00294 | -0.5877 | 1.91E-50 | negative |
| CASP4 | LINC00294 | -0.57363 | 1.30E-47 | negative |
| CASP6 | LINC00294 | -0.61158 | 1.40E-55 | negative |
| CASP8 | LINC00294 | -0.57541 | 5.78E-48 | negative |
| NOD1 | LINC00294 | -0.62926 | 1.13E-59 | negative |
| SCAF11 | AC008115.3 | 0.513452 | 6.42E-37 | postive |
| IL1B | AC245128.3 | 0.592382 | 2.03E-51 | postive |
| NLRP3 | AC245128.3 | 0.530192 | 1.11E-39 | postive |
| CASP1 | AC002480.1 | 0.509109 | 3.16E-36 | postive |
| NLRP2 | AC020907.2 | 0.513498 | 6.31E-37 | postive |
| PRKACA | AC012358.2 | 0.557441 | 1.61E-44 | postive |
| NLRP2 | AC080078.2 | 0.55999 | 5.38E-45 | postive |
| GSDMD | HHLA3 | 0.53112 | 7.73E-40 | postive |
| IL18 | HHLA3 | 0.504896 | 1.45E-35 | postive |
| PYCARD | HHLA3 | 0.584271 | 9.64E-50 | postive |
| PRKACA | AC069228.1 | -0.52029 | 4.99E-38 | negative |
| GSDMB | CD27-AS1 | 0.507954 | 4.80E-36 | postive |
| GSDMB | AC021851.1 | 0.595888 | 3.70E-52 | postive |
| GSDMB | AL590666.1 | 0.570962 | 4.31E-47 | postive |
| NLRP2 | AC025253.1 | 0.555442 | 3.78E-44 | postive |
| NLRP2 | AL020994.2 | 0.541647 | 1.16E-41 | postive |
| CASP6 | GDNF-AS1 | -0.52414 | 1.15E-38 | negative |
| CASP8 | GDNF-AS1 | -0.56818 | 1.49E-46 | negative |
| GSDMD | GDNF-AS1 | -0.50254 | 3.36E-35 | negative |
| PRKACA | GDNF-AS1 | -0.55477 | 5.03E-44 | negative |
| CASP1 | AC022182.2 | 0.618795 | 3.20E-57 | postive |
| CASP4 | AC022182.2 | 0.523295 | 1.59E-38 | postive |
| CASP8 | AC022182.2 | 0.507774 | 5.13E-36 | postive |
| IL18 | AC022182.2 | 0.634944 | 4.75E-61 | postive |
| NLRC4 | AC022182.2 | 0.617518 | 6.29E-57 | postive |
| NLRP3 | AC022182.2 | 0.515886 | 2.60E-37 | postive |
| PYCARD | AC022182.2 | 0.673181 | 4.32E-71 | postive |
| GSDMB | AC245884.8 | 0.667313 | 1.88E-69 | postive |
| PRKACA | AL157700.1 | -0.51511 | 3.47E-37 | negative |
| CASP1 | AC124798.1 | -0.56156 | 2.73E-45 | negative |
| CASP8 | AC124798.1 | -0.56038 | 4.54E-45 | negative |
| IL18 | AC124798.1 | -0.51027 | 2.07E-36 | negative |
| NLRP3 | AC124798.1 | -0.50889 | 3.42E-36 | negative |
| NOD1 | AC124798.1 | -0.54279 | 7.30E-42 | negative |
| GSDMC | LINC01532 | 0.580423 | 5.80E-49 | postive |
| NLRP2 | AL353746.1 | 0.563526 | 1.16E-45 | postive |
| PRKACA | OXCT1-AS1 | -0.55647 | 2.43E-44 | negative |
| GSDMB | AL132655.2 | 0.613371 | 5.52E-56 | postive |
| CASP1 | AC138207.5 | 0.755951 | 4.91E-99 | postive |
| CASP4 | AC138207.5 | 0.614661 | 2.82E-56 | postive |
| CASP8 | AC138207.5 | 0.532122 | 5.22E-40 | postive |
| GSDMD | AC138207.5 | 0.607509 | 1.13E-54 | postive |
| IL18 | AC138207.5 | 0.742125 | 1.26E-93 | postive |
| NLRC4 | AC138207.5 | 0.724726 | 2.72E-87 | postive |
| NLRP3 | AC138207.5 | 0.532641 | 4.25E-40 | postive |
| NOD1 | AC138207.5 | 0.550162 | 3.49E-43 | postive |
| PYCARD | AC138207.5 | 0.863489 | ###### | postive |
| NLRP2 | AC019171.1 | 0.613407 | 5.42E-56 | postive |
| PRKACA | LINC00847 | -0.50407 | 1.95E-35 | negative |
| CASP4 | AL157392.3 | -0.55373 | 7.79E-44 | negative |
| CASP6 | AL157392.3 | -0.57069 | 4.88E-47 | negative |
| GSDMD | AL157392.3 | -0.66555 | 5.73E-69 | negative |
| SCAF11 | AC010624.2 | -0.55506 | 4.45E-44 | negative |
| GSDMB | MCCC1-AS1 | 0.503726 | 2.20E-35 | postive |
| IL18 | LINC00578 | 0.540985 | 1.52E-41 | postive |
| NLRC4 | LINC00578 | 0.55672 | 2.19E-44 | postive |
| NLRP3 | LINC00578 | 0.558691 | 9.42E-45 | postive |
| PJVK | ZNF213-AS1 | 0.540128 | 2.15E-41 | postive |
| NLRP2 | AC022960.2 | 0.535893 | 1.17E-40 | postive |
| IL18 | AC116667.1 | 0.506306 | 8.72E-36 | postive |
| PYCARD | AC116667.1 | 0.514679 | 4.08E-37 | postive |
| GSDMC | LINC00519 | 0.523847 | 1.29E-38 | postive |
| GSDMD | AP003032.1 | 0.51944 | 6.87E-38 | postive |
| CASP1 | DLGAP1-AS1 | 0.519095 | 7.83E-38 | postive |
| NOD1 | DLGAP1-AS1 | 0.514021 | 5.20E-37 | postive |
| PYCARD | DLGAP1-AS1 | 0.540228 | 2.06E-41 | postive |
| PLCG1 | LINC00265 | 0.649659 | 9.63E-65 | postive |
| PLCG1 | AC008737.1 | 0.521502 | 3.15E-38 | postive |
| NLRP2 | LINC02389 | 0.514489 | 4.37E-37 | postive |
| CASP6 | AL441992.1 | 0.505268 | 1.27E-35 | postive |
| GSDMD | AL441992.1 | 0.54345 | 5.58E-42 | postive |
| GSDMB | AC055855.1 | 0.507358 | 5.96E-36 | postive |
| CASP1 | AL606491.1 | 0.537444 | 6.32E-41 | postive |
| IL18 | AL606491.1 | 0.57222 | 2.45E-47 | postive |
| NLRC4 | AL606491.1 | 0.545976 | 1.98E-42 | postive |
| NLRP3 | AL606491.1 | 0.575787 | 4.88E-48 | postive |
| PYCARD | AL606491.1 | 0.649066 | 1.37E-64 | postive |
| CASP1 | AC110995.1 | 0.660526 | 1.32E-67 | postive |
| IL18 | AC110995.1 | 0.589743 | 7.22E-51 | postive |
| NLRC4 | AC110995.1 | 0.709069 | 5.37E-82 | postive |
| NLRP3 | AC110995.1 | 0.599214 | 7.22E-53 | postive |
| PYCARD | AC110995.1 | 0.708125 | 1.09E-81 | postive |
| GPX4 | AC108673.3 | 0.504277 | 1.81E-35 | postive |
| PJVK | AC108673.3 | 0.52024 | 5.08E-38 | postive |
| SCAF11 | AC108673.3 | -0.52044 | 4.72E-38 | negative |
| PLCG1 | AC009065.2 | 0.517817 | 1.27E-37 | postive |
| CASP1 | LINC00339 | 0.521206 | 3.53E-38 | postive |
| IL18 | LINC00339 | 0.513608 | 6.06E-37 | postive |
| PYCARD | LINC00339 | 0.521138 | 3.62E-38 | postive |
| CASP4 | LINC00641 | -0.56346 | 1.19E-45 | negative |
| CASP6 | LINC00641 | -0.61056 | 2.36E-55 | negative |
| GSDMD | LINC00641 | -0.61057 | 2.35E-55 | negative |
| PRKACA | GAS5 | -0.51109 | 1.53E-36 | negative |
| GSDMB | SH3BP5-AS1 | 0.692549 | 8.89E-77 | postive |
| GSDMB | AC078909.2 | 0.655518 | 2.86E-66 | postive |
| PJVK | AC078909.2 | 0.572974 | 1.75E-47 | postive |
| CASP1 | LINC01150 | 0.789679 | ###### | postive |
| CASP4 | LINC01150 | 0.644731 | 1.75E-63 | postive |
| CASP8 | LINC01150 | 0.638055 | 8.18E-62 | postive |
| IL18 | LINC01150 | 0.784332 | ###### | postive |
| NLRC4 | LINC01150 | 0.787225 | ###### | postive |
| NLRP3 | LINC01150 | 0.716696 | 1.57E-84 | postive |
| NOD1 | LINC01150 | 0.61225 | 9.87E-56 | postive |
| PYCARD | LINC01150 | 0.810265 | ###### | postive |
| GSDMB | AC145207.5 | 0.509632 | 2.61E-36 | postive |
| NLRP2 | AC008708.2 | 0.575795 | 4.86E-48 | postive |
| GSDMB | AC009120.2 | 0.668281 | 1.01E-69 | postive |
| PJVK | AC009120.2 | 0.515952 | 2.54E-37 | postive |
| CASP6 | AP001542.3 | 0.526486 | 4.68E-39 | postive |
| GSDMD | AP001542.3 | 0.569508 | 8.26E-47 | postive |
| CASP8 | DICER1-AS1 | -0.55024 | 3.38E-43 | negative |
| PJVK | DICER1-AS1 | 0.659035 | 3.33E-67 | postive |
| CASP4 | MIR4435-2HG | 0.617074 | 7.95E-57 | postive |
| CASP6 | MIR4435-2HG | 0.513661 | 5.94E-37 | postive |
| CASP8 | MIR4435-2HG | 0.500132 | 7.90E-35 | postive |
| CASP8 | AL670729.1 | -0.50523 | 1.28E-35 | negative |
| NLRP2 | AC017076.1 | 0.571643 | 3.18E-47 | postive |
| GSDMB | AC116552.1 | 0.553972 | 7.04E-44 | postive |
| GSDMB | AC005387.1 | 0.625714 | 7.81E-59 | postive |
| GSDMB | AC009955.4 | 0.530325 | 1.06E-39 | postive |
| GSDMB | AP001029.1 | 0.615845 | 1.52E-56 | postive |
| GSDMB | AC020558.2 | 0.6257 | 7.87E-59 | postive |
| CASP9 | AC021739.2 | 0.505813 | 1.04E-35 | postive |
| PRKACA | AC021739.2 | -0.54053 | 1.83E-41 | negative |
| GSDMB | AP001107.4 | 0.524451 | 1.02E-38 | postive |
| GSDMB | SUCLG2-AS1 | -0.50496 | 1.42E-35 | negative |
| PLCG1 | AC104564.3 | 0.509508 | 2.73E-36 | postive |
| GSDMB | AL008582.1 | 0.541429 | 1.27E-41 | postive |
| PJVK | AL008582.1 | 0.570537 | 5.22E-47 | postive |
| GSDMB | AL138960.1 | 0.536409 | 9.56E-41 | postive |
| PJVK | AL138960.1 | 0.613741 | 4.55E-56 | postive |
| PLCG1 | INE1 | 0.504228 | 1.84E-35 | postive |
| CASP6 | SOCS2-AS1 | 0.538473 | 4.19E-41 | postive |
| GSDMD | SOCS2-AS1 | 0.515411 | 3.11E-37 | postive |
| GSDMD | AL117332.1 | 0.525652 | 6.46E-39 | postive |
| CASP1 | PCED1B-AS1 | 0.837425 | ###### | postive |
| CASP4 | PCED1B-AS1 | 0.666727 | 2.72E-69 | postive |
| CASP6 | PCED1B-AS1 | 0.529116 | 1.69E-39 | postive |
| CASP8 | PCED1B-AS1 | 0.61831 | 4.14E-57 | postive |
| GSDMD | PCED1B-AS1 | 0.57708 | 2.70E-48 | postive |
| IL18 | PCED1B-AS1 | 0.853668 | ###### | postive |
| NLRC4 | PCED1B-AS1 | 0.821579 | ###### | postive |
| NLRP3 | PCED1B-AS1 | 0.74979 | 1.40E-96 | postive |
| NOD1 | PCED1B-AS1 | 0.622349 | 4.81E-58 | postive |
| PYCARD | PCED1B-AS1 | 0.904549 | ###### | postive |
| TIRAP | AP001318.2 | 0.555308 | 4.00E-44 | postive |
| CASP6 | AC093726.1 | 0.561931 | 2.32E-45 | postive |
| CASP8 | AC093726.1 | 0.552766 | 1.17E-43 | postive |
| GSDMD | AC093726.1 | 0.625561 | 8.49E-59 | postive |
| CASP1 | AC120036.4 | -0.55281 | 1.15E-43 | negative |
| CASP3 | AC120036.4 | -0.5383 | 4.49E-41 | negative |
| CASP4 | AC120036.4 | -0.61495 | 2.42E-56 | negative |
| CASP6 | AC120036.4 | -0.70892 | 6.01E-82 | negative |
| CASP8 | AC120036.4 | -0.63756 | 1.08E-61 | negative |
| GSDMD | AC120036.4 | -0.58401 | 1.09E-49 | negative |
| IL18 | AC120036.4 | -0.50062 | 6.64E-35 | negative |
| GSDMB | AL031714.1 | 0.636944 | 1.54E-61 | postive |
| PLCG1 | AL031714.1 | 0.545719 | 2.20E-42 | postive |
| CASP1 | AL513217.1 | -0.5305 | 9.86E-40 | negative |
| CASP8 | AL513217.1 | -0.53262 | 4.28E-40 | negative |
| GPX4 | AC005288.1 | -0.52573 | 6.27E-39 | negative |
| SCAF11 | AC005288.1 | 0.571655 | 3.16E-47 | postive |
| CASP1 | AC080038.1 | 0.662418 | 4.09E-68 | postive |
| CASP3 | AC080038.1 | 0.524918 | 8.56E-39 | postive |
| CASP4 | AC080038.1 | 0.687065 | 4.02E-75 | postive |
| CASP6 | AC080038.1 | 0.713874 | 1.39E-83 | postive |
| CASP8 | AC080038.1 | 0.687477 | 3.03E-75 | postive |
| GSDMD | AC080038.1 | 0.670734 | 2.10E-70 | postive |
| IL18 | AC080038.1 | 0.548222 | 7.83E-43 | postive |
| NOD1 | AC080038.1 | 0.619003 | 2.87E-57 | postive |
| PYCARD | AC080038.1 | 0.529946 | 1.22E-39 | postive |
| GSDMD | AL121768.1 | -0.51113 | 1.51E-36 | negative |
| PYCARD | AL121768.1 | -0.53526 | 1.51E-40 | negative |
| CASP4 | MIR600HG | -0.51005 | 2.24E-36 | negative |
| CASP3 | AC064875.1 | 0.514495 | 4.36E-37 | postive |
| GSDMB | RAD51-AS1 | 0.621686 | 6.86E-58 | postive |
| PJVK | RAD51-AS1 | 0.530356 | 1.04E-39 | postive |
| GSDMB | AC069281.2 | 0.715007 | 5.80E-84 | postive |
| GSDMB | AC011468.1 | 0.569452 | 8.47E-47 | postive |
| GSDMB | AL157392.4 | 0.60937 | 4.35E-55 | postive |
| GSDMB | GABPB1-AS1 | 0.613774 | 4.47E-56 | postive |
| GSDMD | LINC01023 | 0.520638 | 4.37E-38 | postive |
| PYCARD | LINC01023 | 0.5053 | 1.25E-35 | postive |
| NLRP2 | AC018358.1 | 0.597658 | 1.56E-52 | postive |
| GSDMB | AC005899.6 | 0.555631 | 3.48E-44 | postive |
| PJVK | AC005899.6 | 0.526133 | 5.37E-39 | postive |
| GPX4 | AC108449.2 | -0.58841 | 1.37E-50 | negative |
| SCAF11 | AC108449.2 | 0.635775 | 2.97E-61 | postive |
| TIRAP | AC108449.2 | 0.501547 | 4.79E-35 | postive |
| GSDMB | AF111167.2 | -0.50197 | 4.12E-35 | negative |
| CASP4 | AC093673.1 | 0.541725 | 1.13E-41 | postive |
| CASP6 | AC093673.1 | 0.557388 | 1.65E-44 | postive |
| GSDMD | AC093673.1 | 0.525716 | 6.30E-39 | postive |
| GSDMB | AL136295.7 | 0.534493 | 2.05E-40 | postive |
| PJVK | AL136295.7 | 0.558828 | 8.88E-45 | postive |
| GSDMB | AC105020.1 | 0.580389 | 5.89E-49 | postive |
| PYCARD | NORAD | -0.61677 | 9.31E-57 | negative |
| GSDMD | AC073896.2 | 0.502233 | 3.75E-35 | postive |
| GSDMB | ARRDC1-AS1 | 0.514015 | 5.21E-37 | postive |
| PJVK | ARRDC1-AS1 | 0.507804 | 5.07E-36 | postive |
| PJVK | AP001160.4 | 0.514832 | 3.85E-37 | postive |
| CASP1 | AC008554.1 | -0.51403 | 5.17E-37 | negative |
| GSDMD | AC008554.1 | -0.71339 | 2.02E-83 | negative |
| IL18 | AC008554.1 | -0.51412 | 5.01E-37 | negative |
| PYCARD | AC008554.1 | -0.61707 | 7.95E-57 | negative |
| TIRAP | AC008554.1 | 0.522469 | 2.18E-38 | postive |
| CASP1 | AL133415.1 | 0.606038 | 2.38E-54 | postive |
| CASP4 | AL133415.1 | 0.578821 | 1.21E-48 | postive |
| CASP6 | AL133415.1 | 0.567836 | 1.74E-46 | postive |
| CASP8 | AL133415.1 | 0.563226 | 1.32E-45 | postive |
| NOD1 | AL133415.1 | 0.577382 | 2.35E-48 | postive |
| GSDMB | AP000866.6 | 0.527493 | 3.17E-39 | postive |
| CASP1 | AC004687.1 | 0.591721 | 2.79E-51 | postive |
| CASP4 | AC004687.1 | 0.512599 | 8.79E-37 | postive |
| GSDMD | AC004687.1 | 0.572178 | 2.50E-47 | postive |
| IL18 | AC004687.1 | 0.592875 | 1.60E-51 | postive |
| NLRC4 | AC004687.1 | 0.531604 | 6.39E-40 | postive |
| PYCARD | AC004687.1 | 0.688954 | 1.09E-75 | postive |
| GSDMB | AC004076.2 | 0.524401 | 1.04E-38 | postive |
| NOD1 | AL359764.1 | -0.51371 | 5.83E-37 | negative |
| NLRP2 | DCTN1-AS1 | 0.562918 | 1.51E-45 | postive |
| GSDMB | LINC00894 | 0.528772 | 1.93E-39 | postive |
| PJVK | LINC00894 | 0.532271 | 4.92E-40 | postive |
| GSDMB | AC097537.1 | 0.503362 | 2.51E-35 | postive |
| GSDMB | BACE1-AS | 0.568304 | 1.41E-46 | postive |
| CASP6 | SLC6A1-AS1 | -0.5007 | 6.47E-35 | negative |
| PYCARD | SLC6A1-AS1 | -0.51019 | 2.13E-36 | negative |
| CASP8 | TDRKH-AS1 | -0.52157 | 3.07E-38 | negative |
| NOD1 | TDRKH-AS1 | -0.51026 | 2.08E-36 | negative |
| GSDMB | AC129510.1 | 0.571619 | 3.21E-47 | postive |
| PLCG1 | AC129510.1 | 0.550936 | 2.53E-43 | postive |
| PJVK | AC007383.2 | 0.538661 | 3.88E-41 | postive |
| NLRP2 | LINC01511 | 0.582019 | 2.76E-49 | postive |
| NLRP2 | AL137786.1 | 0.574598 | 8.37E-48 | postive |
| PLCG1 | POLR2J4 | 0.53727 | 6.78E-41 | postive |
| GSDMB | N4BP2L2-IT2 | 0.610305 | 2.69E-55 | postive |
| PYCARD | AC024075.1 | -0.52011 | 5.34E-38 | negative |
| TIRAP | AC024075.1 | 0.510469 | 1.92E-36 | postive |
| CASP1 | EPB41L4A-AS1 | -0.54271 | 7.55E-42 | negative |
| CASP4 | EPB41L4A-AS1 | -0.54216 | 9.45E-42 | negative |
| CASP6 | EPB41L4A-AS1 | -0.59251 | 1.91E-51 | negative |
| CASP8 | EPB41L4A-AS1 | -0.6624 | 4.14E-68 | negative |
| NOD1 | EPB41L4A-AS1 | -0.52857 | 2.09E-39 | negative |
| PRKACA | EPB41L4A-AS1 | -0.50536 | 1.23E-35 | negative |
| GSDMB | AC099791.2 | 0.579214 | 1.01E-48 | postive |
| PRKACA | LINC00941 | 0.521525 | 3.13E-38 | postive |
| GSDMB | AL355488.1 | 0.727482 | 2.91E-88 | postive |
| CASP1 | AL590764.1 | 0.694077 | 3.03E-77 | postive |
| CASP4 | AL590764.1 | 0.532678 | 4.19E-40 | postive |
| IL18 | AL590764.1 | 0.723091 | 1.01E-86 | postive |
| NLRC4 | AL590764.1 | 0.7485 | 4.47E-96 | postive |
| NLRP3 | AL590764.1 | 0.62772 | 2.61E-59 | postive |
| PYCARD | AL590764.1 | 0.798747 | ###### | postive |
| NLRP2 | LY86-AS1 | 0.613743 | 4.55E-56 | postive |
| CASP1 | AC009119.1 | 0.5791 | 1.07E-48 | postive |
| IL18 | AC009119.1 | 0.61197 | 1.14E-55 | postive |
| NLRC4 | AC009119.1 | 0.710397 | 1.97E-82 | postive |
| NLRP3 | AC009119.1 | 0.545433 | 2.48E-42 | postive |
| PYCARD | AC009119.1 | 0.655992 | 2.14E-66 | postive |
| CASP6 | AL604028.1 | 0.516241 | 2.28E-37 | postive |
| GSDMD | AL604028.1 | 0.543126 | 6.37E-42 | postive |
| PYCARD | AL604028.1 | 0.510322 | 2.03E-36 | postive |
| CASP1 | AC040970.1 | 0.536793 | 8.20E-41 | postive |
| CASP1 | TSC22D1-AS1 | -0.52569 | 6.37E-39 | negative |
| CASP4 | TSC22D1-AS1 | -0.50586 | 1.02E-35 | negative |
| CASP9 | AC013391.3 | 0.508816 | 3.51E-36 | postive |
| GSDMB | AC013391.3 | 0.600466 | 3.88E-53 | postive |
| GSDMB | PTOV1-AS2 | 0.644713 | 1.77E-63 | postive |
| PLCG1 | PTOV1-AS2 | 0.617537 | 6.23E-57 | postive |
| CASP8 | ARHGEF7-AS2 | -0.54132 | 1.33E-41 | negative |
| CASP1 | WARS2-IT1 | 0.516867 | 1.80E-37 | postive |
| NLRC4 | WARS2-IT1 | 0.507335 | 6.01E-36 | postive |
| CASP4 | LNCTAM34A | 0.574678 | 8.07E-48 | postive |
| CASP6 | LNCTAM34A | 0.540074 | 2.20E-41 | postive |
| CASP8 | LNCTAM34A | 0.543328 | 5.87E-42 | postive |
| GSDMD | LNCTAM34A | 0.581162 | 4.11E-49 | postive |
| GSDMB | C8orf44 | 0.555727 | 3.34E-44 | postive |
| PJVK | C8orf44 | 0.511187 | 1.48E-36 | postive |
| GSDMB | AC132938.1 | 0.556209 | 2.72E-44 | postive |
| PJVK | AC132938.1 | 0.503184 | 2.67E-35 | postive |
| GSDMB | AL731563.3 | 0.628534 | 1.67E-59 | postive |
| GPX4 | AC009065.4 | 0.529944 | 1.22E-39 | postive |
| CASP1 | AL022337.1 | -0.51863 | 9.32E-38 | negative |
| CASP8 | AL022337.1 | -0.55154 | 1.96E-43 | negative |
| NOD1 | AL022337.1 | -0.51592 | 2.57E-37 | negative |
| CASP6 | CRNDE | 0.631562 | 3.14E-60 | postive |
| CASP8 | CRNDE | 0.50338 | 2.49E-35 | postive |
| NLRP2 | ANKRD34C-AS1 | 0.610822 | 2.06E-55 | postive |
| IL18 | AC005082.1 | 0.553193 | 9.79E-44 | postive |
| NLRC4 | AC005082.1 | 0.537155 | 7.10E-41 | postive |
| NLRP3 | AC005082.1 | 0.501511 | 4.85E-35 | postive |
| PYCARD | AC005082.1 | 0.589849 | 6.86E-51 | postive |
| GSDMB | AC026471.4 | 0.621682 | 6.87E-58 | postive |
| PJVK | AC026471.4 | 0.529669 | 1.36E-39 | postive |
| GSDMB | AL161452.1 | 0.589531 | 7.99E-51 | postive |
| CASP1 | LINC01160 | 0.524031 | 1.20E-38 | postive |
| GSDMD | LINC01160 | 0.5283 | 2.32E-39 | postive |
| IL18 | LINC01160 | 0.594848 | 6.15E-52 | postive |
| PYCARD | LINC01160 | 0.629679 | 8.91E-60 | postive |
| GPX4 | SCGB1B2P | 0.517526 | 1.41E-37 | postive |
| GSDMB | AC004908.3 | 0.584108 | 1.04E-49 | postive |
| CASP4 | LINC01503 | 0.506302 | 8.73E-36 | postive |
| GSDMD | LINC01503 | 0.552408 | 1.36E-43 | postive |
| GSDMB | AC007292.1 | 0.583994 | 1.10E-49 | postive |
| SCAF11 | AL365205.3 | -0.57198 | 2.74E-47 | negative |
| GSDMB | AC084018.1 | 0.598777 | 8.96E-53 | postive |
| CASP8 | TMCC1-AS1 | -0.52798 | 2.62E-39 | negative |
| PRKACA | TMCC1-AS1 | -0.51323 | 6.96E-37 | negative |
| SCAF11 | STARD4-AS1 | 0.537859 | 5.36E-41 | postive |
| IL6 | MIR22HG | 0.500222 | 7.66E-35 | postive |
| PLCG1 | AL139349.1 | 0.5397 | 2.55E-41 | postive |
| CASP1 | C17orf102 | -0.51131 | 1.41E-36 | negative |
| IL18 | C17orf102 | -0.50638 | 8.49E-36 | negative |
| PYCARD | C17orf102 | -0.52593 | 5.80E-39 | negative |
| CASP6 | AC103974.1 | -0.51338 | 6.58E-37 | negative |
| GSDMD | AC019205.1 | -0.55252 | 1.30E-43 | negative |
| PRKACA | LINC00461 | -0.5125 | 9.11E-37 | negative |
| CASP4 | AL731533.2 | -0.56317 | 1.35E-45 | negative |
| CASP6 | AL731533.2 | -0.52087 | 4.00E-38 | negative |
| CASP8 | AL731533.2 | -0.56625 | 3.50E-46 | negative |
| GSDMB | ASMTL-AS1 | 0.65851 | 4.59E-67 | postive |
| PJVK | ASMTL-AS1 | 0.546982 | 1.31E-42 | postive |
| CASP1 | GAS6-AS1 | 0.581626 | 3.32E-49 | postive |
| CASP4 | GAS6-AS1 | 0.56902 | 1.03E-46 | postive |
| CASP6 | GAS6-AS1 | 0.515939 | 2.55E-37 | postive |
| CASP8 | GAS6-AS1 | 0.618093 | 4.64E-57 | postive |
| GSDMD | GAS6-AS1 | 0.609872 | 3.36E-55 | postive |
| IL18 | GAS6-AS1 | 0.651462 | 3.29E-65 | postive |
| NOD1 | GAS6-AS1 | 0.525602 | 6.58E-39 | postive |
| PYCARD | GAS6-AS1 | 0.574488 | 8.80E-48 | postive |
| GSDMB | SNHG25 | 0.541578 | 1.20E-41 | postive |
| CASP4 | PLBD1-AS1 | 0.526643 | 4.41E-39 | postive |
| CASP8 | PLBD1-AS1 | 0.644834 | 1.65E-63 | postive |
| GSDMB | AC130469.1 | 0.591705 | 2.82E-51 | postive |
| CASP8 | AL118558.3 | -0.5182 | 1.10E-37 | negative |
| NLRP2 | AC080013.1 | 0.560792 | 3.80E-45 | postive |
| GSDMB | SEMA3F-AS1 | 0.646965 | 4.73E-64 | postive |
| IL18 | AL512631.1 | 0.567199 | 2.30E-46 | postive |
| NLRP3 | AL512631.1 | 0.637104 | 1.40E-61 | postive |
| PYCARD | AL512631.1 | 0.582308 | 2.41E-49 | postive |
| CASP6 | TMEM220-AS1 | 0.558573 | 9.90E-45 | postive |
| CASP8 | TMEM220-AS1 | 0.577782 | 1.96E-48 | postive |
| GSDMD | TMEM220-AS1 | 0.619905 | 1.78E-57 | postive |
| CASP1 | AC012213.4 | -0.55469 | 5.19E-44 | negative |
| CASP4 | AC012213.4 | -0.50911 | 3.15E-36 | negative |
| CASP6 | AC012213.4 | -0.55351 | 8.54E-44 | negative |
| CASP8 | AC012213.4 | -0.60072 | 3.43E-53 | negative |
| NOD1 | AC012213.4 | -0.58168 | 3.23E-49 | negative |
| GSDMB | LINC00115 | 0.550023 | 3.70E-43 | postive |
| CASP1 | AL355922.1 | 0.609543 | 3.98E-55 | postive |
| CASP4 | AL355922.1 | 0.51401 | 5.22E-37 | postive |
| IL18 | AL355922.1 | 0.55122 | 2.24E-43 | postive |
| NLRC4 | AL355922.1 | 0.694711 | 1.93E-77 | postive |
| NLRP3 | AL355922.1 | 0.50099 | 5.83E-35 | postive |
| PYCARD | AL355922.1 | 0.580678 | 5.15E-49 | postive |
| CASP6 | AC104985.1 | -0.53478 | 1.83E-40 | negative |
| GSDMD | AC104985.1 | -0.62799 | 2.25E-59 | negative |
| GSDMB | AL133410.1 | 0.565386 | 5.13E-46 | postive |
| PJVK | AL133410.1 | 0.502403 | 3.53E-35 | postive |
| CASP1 | B3GALT5-AS1 | 0.535812 | 1.21E-40 | postive |
| CASP8 | B3GALT5-AS1 | 0.551045 | 2.41E-43 | postive |
| GSDMB | AC103691.1 | 0.562778 | 1.61E-45 | postive |
| PJVK | AC079174.2 | 0.622843 | 3.69E-58 | postive |
| GSDMB | ZKSCAN2-DT | 0.63184 | 2.69E-60 | postive |
| PJVK | ZKSCAN2-DT | 0.526549 | 4.57E-39 | postive |
| GSDMB | AC006435.2 | 0.664072 | 1.45E-68 | postive |
| PLCG1 | AC006435.2 | 0.529349 | 1.54E-39 | postive |
| GSDMB | AC097359.3 | 0.633938 | 8.35E-61 | postive |
| GSDMB | AC005785.1 | 0.734274 | 1.05E-90 | postive |
| PJVK | AC005785.1 | 0.562461 | 1.84E-45 | postive |
| NLRP2 | LINC01574 | 0.533707 | 2.79E-40 | postive |
| GSDMB | LINC01341 | 0.505491 | 1.17E-35 | postive |
| CASP8 | AC008124.1 | -0.52362 | 1.41E-38 | negative |
| PRKACA | AC008124.1 | -0.53502 | 1.66E-40 | negative |
| GSDMB | LINC00106 | 0.501012 | 5.79E-35 | postive |
| CASP4 | LINC02283 | -0.50543 | 1.19E-35 | negative |
| CASP6 | LINC02283 | -0.60959 | 3.88E-55 | negative |
| CASP8 | LINC02283 | -0.61125 | 1.66E-55 | negative |
| GSDMD | LINC02283 | -0.53606 | 1.10E-40 | negative |
| PRKACA | LINC02283 | -0.57049 | 5.34E-47 | negative |
| GSDMB | AC020907.4 | 0.608517 | 6.73E-55 | postive |
| PLCG1 | AC020907.4 | 0.505568 | 1.14E-35 | postive |
| CASP1 | AC026790.1 | -0.5278 | 2.82E-39 | negative |
| CASP4 | AC026790.1 | -0.50485 | 1.47E-35 | negative |
| NOD1 | AC026790.1 | -0.55645 | 2.46E-44 | negative |
| CASP1 | LINC00324 | 0.579142 | 1.05E-48 | postive |
| CASP4 | LINC00324 | 0.513728 | 5.80E-37 | postive |
| IL18 | LINC00324 | 0.524173 | 1.14E-38 | postive |
| NLRC4 | LINC00324 | 0.559146 | 7.74E-45 | postive |
| PYCARD | LINC00324 | 0.546394 | 1.67E-42 | postive |
| CASP6 | AL035530.2 | -0.52316 | 1.68E-38 | negative |
| CASP8 | AL035530.2 | -0.52603 | 5.59E-39 | negative |
| GSDMD | AL035530.2 | -0.58387 | 1.16E-49 | negative |
| GSDMD | AL021368.2 | -0.50039 | 7.20E-35 | negative |
| PRKACA | AL021368.2 | -0.58494 | 7.05E-50 | negative |
| GSDMB | AL031705.1 | 0.607982 | 8.85E-55 | postive |
| CASP1 | AC018755.4 | 0.604885 | 4.25E-54 | postive |
| IL18 | AC018755.4 | 0.706469 | 3.77E-81 | postive |
| NLRC4 | AC018755.4 | 0.624758 | 1.31E-58 | postive |
| NLRP3 | AC018755.4 | 0.556305 | 2.61E-44 | postive |
| PYCARD | AC018755.4 | 0.684412 | 2.47E-74 | postive |
| GSDMB | AL606760.2 | 0.523567 | 1.44E-38 | postive |
| NLRP2 | AC103855.2 | 0.588221 | 1.49E-50 | postive |
| GSDMB | ZNF32-AS2 | 0.520238 | 5.09E-38 | postive |
| CASP4 | AL118505.1 | -0.50726 | 6.16E-36 | negative |
| CASP6 | AL118505.1 | -0.56047 | 4.38E-45 | negative |
| CASP8 | AL118505.1 | -0.56242 | 1.87E-45 | negative |
| CASP9 | AL118505.1 | 0.546531 | 1.58E-42 | postive |
| GSDMD | AL118505.1 | -0.55113 | 2.33E-43 | negative |
| PRKACA | AL118505.1 | -0.5532 | 9.75E-44 | negative |
| GSDMB | AP001160.1 | 0.672848 | 5.36E-71 | postive |
| CASP9 | LINC00689 | 0.635343 | 3.79E-61 | postive |
| NLRP2 | AC103681.2 | 0.501021 | 5.77E-35 | postive |
| NLRP2 | JAZF1-AS1 | 0.546314 | 1.72E-42 | postive |
| GSDMB | AC011481.2 | 0.66216 | 4.80E-68 | postive |
| CASP6 | AL512625.2 | -0.50498 | 1.41E-35 | negative |
| GSDMD | AL512625.2 | -0.60619 | 2.20E-54 | negative |
| GSDMB | LINC01772 | 0.619985 | 1.70E-57 | postive |
| GSDMD | ACVR2B-AS1 | -0.53031 | 1.06E-39 | negative |
| GSDMB | TPT1-AS1 | 0.601267 | 2.61E-53 | postive |
| GSDMB | CTBP1-AS | 0.600359 | 4.09E-53 | postive |
| PLCG1 | CTBP1-AS | 0.500484 | 6.98E-35 | postive |
| NOD1 | AC124319.1 | 0.522791 | 1.93E-38 | postive |
| GSDMB | AC005332.5 | 0.536802 | 8.17E-41 | postive |
| PJVK | AL359881.1 | 0.543205 | 6.17E-42 | postive |
| PJVK | PARD3-AS1 | 0.562873 | 1.54E-45 | postive |
| GSDMB | AC027796.4 | 0.508011 | 4.70E-36 | postive |
| CASP1 | AC008760.2 | 0.738618 | 2.62E-92 | postive |
| CASP4 | AC008760.2 | 0.720409 | 8.52E-86 | postive |
| CASP6 | AC008760.2 | 0.549041 | 5.57E-43 | postive |
| CASP8 | AC008760.2 | 0.667377 | 1.80E-69 | postive |
| GSDMD | AC008760.2 | 0.529808 | 1.29E-39 | postive |
| IL18 | AC008760.2 | 0.659634 | 2.30E-67 | postive |
| NLRC4 | AC008760.2 | 0.719055 | 2.48E-85 | postive |
| NOD1 | AC008760.2 | 0.570729 | 4.79E-47 | postive |
| PYCARD | AC008760.2 | 0.633379 | 1.14E-60 | postive |
| GSDMB | AC027601.3 | 0.619138 | 2.67E-57 | postive |
| PRKACA | AL162231.2 | 0.510475 | 1.92E-36 | postive |
| SCAF11 | AC073046.1 | 0.598222 | 1.18E-52 | postive |
| CASP8 | AC012645.1 | -0.52308 | 1.73E-38 | negative |
| GSDMB | AL162586.1 | 0.529549 | 1.43E-39 | postive |
| CASP1 | PAXBP1-AS1 | -0.58705 | 2.60E-50 | negative |
| CASP4 | PAXBP1-AS1 | -0.58768 | 1.93E-50 | negative |
| CASP6 | PAXBP1-AS1 | -0.56096 | 3.54E-45 | negative |
| CASP8 | PAXBP1-AS1 | -0.56187 | 2.39E-45 | negative |
| GSDMD | PAXBP1-AS1 | -0.55717 | 1.81E-44 | negative |
| IL18 | PAXBP1-AS1 | -0.50069 | 6.50E-35 | negative |
| NOD1 | PAXBP1-AS1 | -0.50012 | 7.95E-35 | negative |
| PYCARD | PAXBP1-AS1 | -0.52186 | 2.76E-38 | negative |
| CASP4 | AC004803.1 | -0.54511 | 2.82E-42 | negative |
| CASP6 | AC004803.1 | -0.54683 | 1.39E-42 | negative |
| CASP8 | AC004803.1 | -0.55046 | 3.08E-43 | negative |
| GSDMD | AC004803.1 | -0.60357 | 8.26E-54 | negative |
| SCAF11 | AP001160.3 | -0.5775 | 2.23E-48 | negative |
| PJVK | AC005899.7 | 0.530331 | 1.05E-39 | postive |
| GPX4 | EBLN3P | -0.51408 | 5.09E-37 | negative |
| PYCARD | EBLN3P | -0.54013 | 2.15E-41 | negative |
| GSDMB | THUMPD3-AS1 | 0.688034 | 2.06E-75 | postive |
| GSDMB | AC073869.1 | 0.736281 | 1.92E-91 | postive |
| CASP8 | KCNIP2-AS1 | -0.50443 | 1.71E-35 | negative |
| GSDMB | AL139287.1 | 0.703113 | 4.50E-80 | postive |
| GSDMB | AC005393.1 | 0.574061 | 1.07E-47 | postive |
| CASP1 | AC147067.1 | 0.634379 | 6.52E-61 | postive |
| CASP4 | AC147067.1 | 0.602365 | 1.51E-53 | postive |
| GSDMD | AC147067.1 | 0.58194 | 2.87E-49 | postive |
| IL18 | AC147067.1 | 0.66648 | 3.18E-69 | postive |
| NLRC4 | AC147067.1 | 0.591173 | 3.64E-51 | postive |
| NOD1 | AC147067.1 | 0.50121 | 5.40E-35 | postive |
| PYCARD | AC147067.1 | 0.747891 | 7.71E-96 | postive |
| GSDMD | AC002398.1 | 0.503155 | 2.70E-35 | postive |
| PYCARD | AC002398.1 | 0.507599 | 5.46E-36 | postive |
| GSDMB | AC010326.3 | 0.545887 | 2.05E-42 | postive |
| PRKACA | AC079766.1 | 0.511803 | 1.18E-36 | postive |
| CASP1 | BASP1-AS1 | -0.62141 | 7.95E-58 | negative |
| CASP4 | BASP1-AS1 | -0.54814 | 8.11E-43 | negative |
| CASP6 | BASP1-AS1 | -0.50618 | 9.11E-36 | negative |
| CASP8 | BASP1-AS1 | -0.52187 | 2.74E-38 | negative |
| IL18 | BASP1-AS1 | -0.55785 | 1.35E-44 | negative |
| NOD1 | BASP1-AS1 | -0.59539 | 4.72E-52 | negative |
| PYCARD | BASP1-AS1 | -0.54802 | 8.52E-43 | negative |
| CASP6 | AC053503.1 | -0.58091 | 4.62E-49 | negative |
| CASP8 | AC053503.1 | -0.58389 | 1.16E-49 | negative |
| PRKACA | AC053503.1 | -0.51348 | 6.35E-37 | negative |
| PLCG1 | AL390728.4 | 0.527048 | 3.77E-39 | postive |
| SCAF11 | AL390728.4 | 0.594425 | 7.55E-52 | postive |
| CASP1 | NDUFA6-DT | -0.64059 | 1.92E-62 | negative |
| CASP4 | NDUFA6-DT | -0.61543 | 1.88E-56 | negative |
| CASP6 | NDUFA6-DT | -0.6133 | 5.72E-56 | negative |
| CASP8 | NDUFA6-DT | -0.58481 | 7.50E-50 | negative |
| GSDMD | NDUFA6-DT | -0.64102 | 1.50E-62 | negative |
| IL18 | NDUFA6-DT | -0.62888 | 1.38E-59 | negative |
| PYCARD | NDUFA6-DT | -0.59547 | 4.55E-52 | negative |
| CASP1 | FAM66C | -0.50933 | 2.91E-36 | negative |
| GSDMD | FAM66C | -0.50681 | 7.27E-36 | negative |
| PYCARD | FAM66C | -0.5003 | 7.45E-35 | negative |
| CASP4 | LINC01871 | 0.505414 | 1.20E-35 | postive |
| GSDMB | SNHG3 | 0.60528 | 3.49E-54 | postive |
| GSDMB | AC048341.2 | 0.60013 | 4.59E-53 | postive |
| GSDMB | AC005261.1 | 0.50778 | 5.12E-36 | postive |
| CASP1 | AC023794.4 | 0.633764 | 9.21E-61 | postive |
| CASP4 | AC023794.4 | 0.514393 | 4.53E-37 | postive |
| CASP8 | AC023794.4 | 0.557639 | 1.48E-44 | postive |
| IL18 | AC023794.4 | 0.671194 | 1.56E-70 | postive |
| NLRC4 | AC023794.4 | 0.599711 | 5.65E-53 | postive |
| PYCARD | AC023794.4 | 0.684597 | 2.17E-74 | postive |
| IL18 | AL158071.4 | 0.635824 | 2.89E-61 | postive |
| PYCARD | AL158071.4 | 0.667233 | 1.97E-69 | postive |
| NLRP2 | AC110491.1 | 0.505675 | 1.09E-35 | postive |
| GSDMD | AC105206.2 | -0.50382 | 2.13E-35 | negative |
| SCAF11 | AL732292.2 | -0.54496 | 3.01E-42 | negative |
| CASP6 | AC010226.1 | 0.575758 | 4.94E-48 | postive |
| CASP8 | AC010226.1 | 0.549129 | 5.37E-43 | postive |
| GSDMD | AC010226.1 | 0.603148 | 1.02E-53 | postive |
| SCAF11 | AC019080.1 | 0.500425 | 7.13E-35 | postive |
| GSDMB | AC095057.3 | 0.572017 | 2.69E-47 | postive |
| GPX4 | AC010834.3 | -0.50754 | 5.57E-36 | negative |
| GSDMD | AC010834.3 | -0.53943 | 2.85E-41 | negative |
| SCAF11 | AC010834.3 | 0.588878 | 1.09E-50 | postive |
| SCAF11 | AC253536.3 | 0.564373 | 8.00E-46 | postive |
| GSDMB | AC009133.1 | 0.571624 | 3.20E-47 | postive |
| GSDMB | AC018638.7 | 0.558595 | 9.81E-45 | postive |
| CASP9 | AL133304.3 | 0.53463 | 1.94E-40 | postive |
| CASP6 | AC080038.2 | 0.531383 | 6.97E-40 | postive |
| GSDMD | AC080038.2 | 0.547678 | 9.81E-43 | postive |
| CASP8 | AL157400.3 | -0.50052 | 6.89E-35 | negative |
| GSDMB | AC021739.3 | 0.511759 | 1.20E-36 | postive |
| GSDMB | AC025178.1 | 0.592036 | 2.40E-51 | postive |
| NLRP2 | LINC00622 | 0.507039 | 6.69E-36 | postive |
| NLRP3 | LINC02642 | 0.639683 | 3.23E-62 | postive |
| CASP1 | AC007728.2 | 0.561973 | 2.28E-45 | postive |
| CASP4 | AC007728.2 | 0.540321 | 1.99E-41 | postive |
| CASP8 | AC007728.2 | 0.500996 | 5.82E-35 | postive |
| NLRC4 | AC007728.2 | 0.550713 | 2.77E-43 | postive |
| CASP9 | AL021408.1 | 0.520368 | 4.84E-38 | postive |
| PRKACA | AC092338.1 | 0.628994 | 1.30E-59 | postive |
| SCAF11 | AP001972.1 | -0.50817 | 4.44E-36 | negative |
| CASP1 | AC087273.2 | 0.559684 | 6.14E-45 | postive |
| IL18 | AC087273.2 | 0.667045 | 2.23E-69 | postive |
| NLRC4 | AC087273.2 | 0.612104 | 1.06E-55 | postive |
| NLRP3 | AC087273.2 | 0.565924 | 4.05E-46 | postive |
| PYCARD | AC087273.2 | 0.686056 | 8.03E-75 | postive |
| GSDMB | LINC01355 | 0.74748 | 1.11E-95 | postive |
| NLRP2 | AC134312.3 | 0.550735 | 2.75E-43 | postive |
| GSDMC | LINC00906 | 0.520264 | 5.04E-38 | postive |
| CASP1 | AL133371.2 | 0.646141 | 7.68E-64 | postive |
| IL18 | AL133371.2 | 0.737134 | 9.31E-92 | postive |
| NLRC4 | AL133371.2 | 0.661767 | 6.13E-68 | postive |
| NLRP3 | AL133371.2 | 0.682909 | 6.83E-74 | postive |
| PYCARD | AL133371.2 | 0.660613 | 1.26E-67 | postive |
| NLRP2 | AC138649.1 | 0.534946 | 1.71E-40 | postive |
| GSDMD | AC007663.4 | -0.5442 | 4.11E-42 | negative |
| GSDMB | STAG3L5P-PVRIG2P-PILRB | 0.529929 | 1.23E-39 | postive |
| CASP6 | AL353796.1 | -0.50191 | 4.21E-35 | negative |
| PRKACA | SNHG29 | -0.5306 | 9.47E-40 | negative |
| NLRP2 | AP003721.1 | 0.520443 | 4.71E-38 | postive |
| GSDMB | SNHG6 | 0.505753 | 1.06E-35 | postive |
| CASP8 | AC022762.2 | -0.50319 | 2.67E-35 | negative |
| CASP4 | AC005070.3 | -0.50068 | 6.52E-35 | negative |
| GSDMB | SNHG12 | 0.703456 | 3.50E-80 | postive |
| GSDMB | AC007038.1 | 0.63404 | 7.89E-61 | postive |
| CASP4 | Z97653.1 | -0.5285 | 2.14E-39 | negative |
| CASP8 | Z97653.1 | -0.55887 | 8.72E-45 | negative |
| GSDMB | AC090589.3 | 0.514395 | 4.53E-37 | postive |
| GSDMB | AC015813.4 | 0.549174 | 5.27E-43 | postive |
| GSDMB | AC004449.1 | 0.501513 | 4.85E-35 | postive |
| CASP8 | Z95115.1 | -0.52479 | 9.01E-39 | negative |
| GSDMB | ARHGAP27P1-BPTFP1-KPNA2P3 | 0.731055 | 1.54E-89 | postive |
| PJVK | ARHGAP27P1-BPTFP1-KPNA2P3 | 0.517573 | 1.39E-37 | postive |
| SCAF11 | AC018752.1 | 0.503114 | 2.74E-35 | postive |
| SCAF11 | AL844908.2 | -0.51703 | 1.70E-37 | negative |
| GSDMB | Z97832.2 | 0.666473 | 3.20E-69 | postive |
| NLRP2 | SLC26A4-AS1 | 0.5568 | 2.12E-44 | postive |
| CASP8 | NRAV | 0.508413 | 4.07E-36 | postive |
| GSDMD | AL645933.2 | 0.555884 | 3.13E-44 | postive |
| NOD1 | SILC1 | -0.51722 | 1.58E-37 | negative |
| NLRP2 | AC015712.2 | 0.576224 | 3.99E-48 | postive |
| CASP1 | OIP5-AS1 | -0.50449 | 1.67E-35 | negative |
| PYCARD | OIP5-AS1 | -0.56883 | 1.12E-46 | negative |
| NLRP2 | AC066612.2 | 0.574045 | 1.08E-47 | postive |
| CASP1 | MIR155HG | 0.552863 | 1.12E-43 | postive |
| CASP4 | MIR155HG | 0.678798 | 1.07E-72 | postive |
| CASP6 | MIR155HG | 0.612247 | 9.89E-56 | postive |
| CASP8 | MIR155HG | 0.608883 | 5.58E-55 | postive |
| GSDMD | MIR155HG | 0.650539 | 5.71E-65 | postive |
| GSDMB | AL512770.1 | 0.692738 | 7.79E-77 | postive |
| GSDMB | AC135050.3 | 0.554707 | 5.16E-44 | postive |
| PJVK | AC135050.3 | 0.521599 | 3.04E-38 | postive |
| GSDMB | LINC01786 | 0.626267 | 5.78E-59 | postive |
| GSDMB | ELF3-AS1 | 0.619728 | 1.95E-57 | postive |
| NLRP2 | AL049651.2 | 0.544123 | 4.24E-42 | postive |
| GSDMB | SNHG10 | 0.536739 | 8.38E-41 | postive |
| GSDMB | AC253576.2 | 0.517954 | 1.20E-37 | postive |
| PJVK | AL590096.1 | 0.563098 | 1.40E-45 | postive |
| CASP6 | AP002840.2 | 0.504873 | 1.46E-35 | postive |
| CASP8 | AP002840.2 | 0.530132 | 1.14E-39 | postive |
| GSDMD | AP002840.2 | 0.657905 | 6.66E-67 | postive |
| GSDMB | AC016747.3 | 0.524086 | 1.18E-38 | postive |
| CASP4 | AC008669.1 | -0.52738 | 3.31E-39 | negative |
| CASP6 | AC008669.1 | -0.51316 | 7.14E-37 | negative |
| CASP8 | AC008669.1 | -0.53115 | 7.63E-40 | negative |
| GSDMD | AC008669.1 | -0.5062 | 9.05E-36 | negative |
| GSDMD | AC245060.2 | -0.50334 | 2.53E-35 | negative |
| PYCARD | AC245060.2 | -0.50484 | 1.48E-35 | negative |
| SCAF11 | AC245060.2 | 0.531756 | 6.02E-40 | postive |
| CASP1 | L3MBTL4-AS1 | 0.7572 | 1.53E-99 | postive |
| CASP4 | L3MBTL4-AS1 | 0.627146 | 3.58E-59 | postive |
| CASP8 | L3MBTL4-AS1 | 0.627625 | 2.75E-59 | postive |
| GSDMD | L3MBTL4-AS1 | 0.543408 | 5.68E-42 | postive |
| IL18 | L3MBTL4-AS1 | 0.816906 | ###### | postive |
| NLRC4 | L3MBTL4-AS1 | 0.728941 | 8.80E-89 | postive |
| NLRP3 | L3MBTL4-AS1 | 0.654571 | 5.07E-66 | postive |
| NOD1 | L3MBTL4-AS1 | 0.532918 | 3.81E-40 | postive |
| PYCARD | L3MBTL4-AS1 | 0.805459 | ###### | postive |
| GPX4 | AC109322.1 | 0.513987 | 5.27E-37 | postive |
| NLRP2 | LINC01164 | 0.522029 | 2.58E-38 | postive |
| PYCARD | AC025917.1 | -0.50014 | 7.89E-35 | negative |
| SCAF11 | AC025917.1 | 0.53323 | 3.37E-40 | postive |
| GSDMB | LINC00174 | 0.584224 | 9.86E-50 | postive |
| PLCG1 | LINC00174 | 0.532171 | 5.12E-40 | postive |
| GSDMB | AL928654.2 | 0.584556 | 8.43E-50 | postive |
| NLRP2 | LINC00398 | 0.502346 | 3.60E-35 | postive |
| CASP1 | AC011899.2 | 0.794244 | ###### | postive |
| CASP4 | AC011899.2 | 0.768472 | ###### | postive |
| CASP6 | AC011899.2 | 0.574858 | 7.44E-48 | postive |
| CASP8 | AC011899.2 | 0.708496 | 8.27E-82 | postive |
| GSDMD | AC011899.2 | 0.577823 | 1.92E-48 | postive |
| IL18 | AC011899.2 | 0.759356 | ###### | postive |
| NLRC4 | AC011899.2 | 0.789653 | ###### | postive |
| NOD1 | AC011899.2 | 0.604819 | 4.40E-54 | postive |
| PYCARD | AC011899.2 | 0.746123 | 3.73E-95 | postive |
| SCAF11 | IBA57-DT | -0.53135 | 7.08E-40 | negative |
| CASP1 | AC009948.1 | -0.51187 | 1.15E-36 | negative |
| NLRP3 | AC009948.1 | -0.52018 | 5.19E-38 | negative |
| NOD1 | AC009948.1 | -0.50954 | 2.70E-36 | negative |
| CASP1 | AC254562.3 | -0.51808 | 1.15E-37 | negative |
| CASP4 | AC254562.3 | -0.58809 | 1.59E-50 | negative |
| CASP6 | AC254562.3 | -0.5099 | 2.36E-36 | negative |
| CASP8 | AC254562.3 | -0.66832 | 9.86E-70 | negative |
| CASP8 | AC025211.1 | -0.53133 | 7.11E-40 | negative |
| PRKACA | AC025211.1 | -0.57975 | 7.90E-49 | negative |
| NLRP2 | LINC00507 | 0.537767 | 5.56E-41 | postive |
| GPX4 | AL035071.1 | 0.502383 | 3.56E-35 | postive |
| GSDMB | AC012236.1 | 0.514914 | 3.73E-37 | postive |
| CASP1 | MIR7-3HG | -0.5139 | 5.43E-37 | negative |
| NOD1 | MIR7-3HG | -0.54033 | 1.99E-41 | negative |
| GPX4 | Z68871.1 | -0.53315 | 3.49E-40 | negative |
| PYCARD | Z68871.1 | -0.50629 | 8.76E-36 | negative |
| SCAF11 | Z68871.1 | 0.551347 | 2.13E-43 | postive |
| GSDMB | AC005306.1 | 0.626422 | 5.31E-59 | postive |
| SCAF11 | AL022328.3 | -0.53465 | 1.93E-40 | negative |
| SCAF11 | AC027682.6 | -0.59326 | 1.33E-51 | negative |
| GSDMD | AL450270.1 | 0.514727 | 4.00E-37 | postive |
| GSDMB | AL356481.3 | 0.584952 | 7.00E-50 | postive |
| CASP1 | AL353751.1 | -0.52959 | 1.41E-39 | negative |
| CASP4 | AL353751.1 | -0.52178 | 2.83E-38 | negative |
| CASP6 | AL353751.1 | -0.53524 | 1.52E-40 | negative |
| CASP8 | AL353751.1 | -0.56331 | 1.27E-45 | negative |
| NOD1 | AL353751.1 | -0.50523 | 1.28E-35 | negative |
| GSDMB | AC127024.6 | 0.66642 | 3.31E-69 | postive |
| PJVK | AC127024.6 | 0.512673 | 8.55E-37 | postive |
| NLRP2 | LINC01202 | 0.590842 | 4.26E-51 | postive |
| GSDMB | AC010300.1 | 0.561358 | 2.98E-45 | postive |
| NLRP2 | AC010857.1 | 0.519487 | 6.75E-38 | postive |
| GSDMB | AC008735.1 | 0.510911 | 1.63E-36 | postive |
| PLCG1 | AC008735.1 | 0.521917 | 2.69E-38 | postive |
| GSDMB | LINC00685 | 0.591331 | 3.37E-51 | postive |
| PJVK | LINC00685 | 0.527174 | 3.59E-39 | postive |
| CASP9 | LINC00609 | 0.507677 | 5.31E-36 | postive |
| GSDMB | LINC00609 | 0.516519 | 2.06E-37 | postive |
| GSDMB | LINC01004 | 0.619541 | 2.15E-57 | postive |
| PJVK | LINC01004 | 0.502857 | 3.00E-35 | postive |
| GSDMB | AC092794.2 | 0.538647 | 3.90E-41 | postive |
| GSDMB | AP006545.2 | 0.572206 | 2.47E-47 | postive |
| CASP1 | LINC01480 | 0.547139 | 1.23E-42 | postive |
| IL18 | LINC01480 | 0.669342 | 5.14E-70 | postive |
| NLRC4 | LINC01480 | 0.530742 | 8.97E-40 | postive |
| NLRP3 | LINC01480 | 0.629118 | 1.21E-59 | postive |
| PYCARD | LINC01480 | 0.692666 | 8.19E-77 | postive |
| TNF | LINC01480 | 0.520537 | 4.54E-38 | postive |
| CASP6 | AC026401.3 | 0.556861 | 2.06E-44 | postive |
| GSDMD | AC026401.3 | 0.510871 | 1.66E-36 | postive |
| GSDMD | CA3-AS1 | 0.523185 | 1.66E-38 | postive |
| NOD1 | AC121493.1 | -0.50941 | 2.83E-36 | negative |
| CASP1 | AL135999.3 | -0.56168 | 2.59E-45 | negative |
| CASP6 | AL135999.3 | -0.52648 | 4.70E-39 | negative |
| IL18 | AL135999.3 | -0.54151 | 1.23E-41 | negative |
| NOD1 | AL135999.3 | -0.5465 | 1.60E-42 | negative |
| PYCARD | AL135999.3 | -0.51846 | 9.94E-38 | negative |
| CASP1 | AC099521.1 | -0.50555 | 1.15E-35 | negative |
| CASP4 | AC099521.1 | -0.51391 | 5.43E-37 | negative |
| CASP8 | AC099521.1 | -0.56028 | 4.74E-45 | negative |
| GSDMD | AC099521.1 | -0.55235 | 1.40E-43 | negative |
| NLRP2 | LINC01018 | 0.549808 | 4.05E-43 | postive |
| TIRAP | RBM26-AS1 | 0.501021 | 5.77E-35 | postive |
| CASP8 | AL589765.6 | -0.58626 | 3.78E-50 | negative |
| NOD1 | AL589765.6 | -0.54754 | 1.04E-42 | negative |
| PRKACA | LINC01637 | 0.555805 | 3.24E-44 | postive |
| GSDMD | AC011477.3 | -0.5406 | 1.78E-41 | negative |
| NLRP2 | AC139491.1 | 0.600332 | 4.15E-53 | postive |
| NLRP2 | AC134312.1 | 0.571296 | 3.71E-47 | postive |
| GSDMB | AC004263.1 | 0.596792 | 2.38E-52 | postive |
| NLRP2 | LINC01616 | 0.596119 | 3.31E-52 | postive |
| GSDMB | AC027601.1 | 0.621396 | 8.01E-58 | postive |
| GSDMD | AC126407.1 | -0.5423 | 8.92E-42 | negative |
| GSDMB | AP006623.1 | 0.609225 | 4.69E-55 | postive |
| PLCG1 | AC011005.4 | 0.584926 | 7.09E-50 | postive |
| GSDMB | AC245060.6 | 0.556697 | 2.21E-44 | postive |
| NLRP2 | AC104024.2 | 0.615266 | 2.05E-56 | postive |
| GSDMB | AC005387.2 | 0.641008 | 1.51E-62 | postive |
| TIRAP | FAM222A-AS1 | 0.524033 | 1.20E-38 | postive |
| GSDMD | HCG18 | -0.57554 | 5.46E-48 | negative |
| IL18 | HCG18 | -0.50683 | 7.21E-36 | negative |
| PYCARD | HCG18 | -0.57104 | 4.16E-47 | negative |
| CASP1 | AC138207.4 | 0.701375 | 1.61E-79 | postive |
| CASP4 | AC138207.4 | 0.656166 | 1.92E-66 | postive |
| CASP6 | AC138207.4 | 0.508111 | 4.54E-36 | postive |
| CASP8 | AC138207.4 | 0.591412 | 3.24E-51 | postive |
| GSDMD | AC138207.4 | 0.542198 | 9.29E-42 | postive |
| IL18 | AC138207.4 | 0.609982 | 3.18E-55 | postive |
| NLRC4 | AC138207.4 | 0.62782 | 2.47E-59 | postive |
| NOD1 | AC138207.4 | 0.60045 | 3.91E-53 | postive |
| PYCARD | AC138207.4 | 0.63029 | 6.36E-60 | postive |
| CASP4 | AC127502.2 | 0.512371 | 9.56E-37 | postive |
| CASP6 | AC127502.2 | 0.557802 | 1.38E-44 | postive |
| CASP8 | AC127502.2 | 0.506977 | 6.84E-36 | postive |
| GSDMD | AC127502.2 | 0.555214 | 4.16E-44 | postive |
| GSDMB | LINC00342 | 0.686477 | 6.02E-75 | postive |
| PJVK | LINC00342 | 0.511539 | 1.30E-36 | postive |
| GSDMB | AC116914.2 | 0.619837 | 1.84E-57 | postive |
| SCAF11 | AC006213.1 | 0.554222 | 6.34E-44 | postive |
| CASP1 | AL357033.4 | 0.597944 | 1.35E-52 | postive |
| CASP4 | AL357033.4 | 0.70266 | 6.27E-80 | postive |
| CASP8 | AL357033.4 | 0.607502 | 1.13E-54 | postive |
| GSDMD | AL357033.4 | 0.578836 | 1.21E-48 | postive |
| IL18 | AL357033.4 | 0.528697 | 1.99E-39 | postive |
| NLRC4 | AL357033.4 | 0.519739 | 6.14E-38 | postive |
| PYCARD | AL357033.4 | 0.507283 | 6.12E-36 | postive |
| CASP6 | AC015922.3 | 0.525541 | 6.74E-39 | postive |
| CASP8 | AC015922.3 | 0.515292 | 3.25E-37 | postive |
| GSDMD | AC015922.3 | 0.537397 | 6.44E-41 | postive |
| PRKACA | AC007950.1 | -0.52824 | 2.38E-39 | negative |
| CASP4 | AC009113.1 | -0.53077 | 8.88E-40 | negative |
| CASP8 | AC009113.1 | -0.51764 | 1.35E-37 | negative |
| PJVK | AC003102.1 | 0.530715 | 9.06E-40 | postive |
| GSDMB | LINC00173 | 0.548951 | 5.78E-43 | postive |
| PJVK | LINC00173 | 0.527825 | 2.79E-39 | postive |
| GSDMB | AC006111.3 | 0.502198 | 3.80E-35 | postive |
| GSDMB | AL022328.2 | 0.696156 | 6.92E-78 | postive |
| CASP1 | AC139530.1 | 0.680483 | 3.49E-73 | postive |
| CASP4 | AC139530.1 | 0.679082 | 8.90E-73 | postive |
| CASP8 | AC139530.1 | 0.525674 | 6.40E-39 | postive |
| GSDMD | AC139530.1 | 0.516834 | 1.83E-37 | postive |
| IL18 | AC139530.1 | 0.637725 | 9.86E-62 | postive |
| NLRC4 | AC139530.1 | 0.6567 | 1.39E-66 | postive |
| NOD1 | AC139530.1 | 0.519933 | 5.71E-38 | postive |
| PYCARD | AC139530.1 | 0.67935 | 7.44E-73 | postive |
| CASP1 | AC092720.2 | -0.55853 | 1.01E-44 | negative |
| CASP4 | AC092720.2 | -0.54166 | 1.15E-41 | negative |
| CASP6 | AC092720.2 | -0.51736 | 1.50E-37 | negative |
| CASP8 | AC092720.2 | -0.52653 | 4.60E-39 | negative |
| NOD1 | AC092720.2 | -0.59903 | 7.91E-53 | negative |
| GSDMB | AL391425.1 | 0.607402 | 1.19E-54 | postive |
| PRKACA | NNT-AS1 | -0.54099 | 1.51E-41 | negative |
| CASP8 | AP000757.1 | -0.56221 | 2.06E-45 | negative |
| GSDMB | AP000757.1 | 0.518278 | 1.06E-37 | postive |
| PRKACA | AP000757.1 | -0.53544 | 1.41E-40 | negative |
| CASP1 | AL121827.1 | -0.58375 | 1.23E-49 | negative |
| CASP4 | AL121827.1 | -0.50253 | 3.37E-35 | negative |
| CASP6 | AL121827.1 | -0.50497 | 1.41E-35 | negative |
| IL18 | AL121827.1 | -0.52947 | 1.47E-39 | negative |
| PYCARD | AL121827.1 | -0.50224 | 3.74E-35 | negative |
| CASP1 | TGFB2-AS1 | 0.512341 | 9.67E-37 | postive |
| CASP6 | TGFB2-AS1 | 0.508165 | 4.45E-36 | postive |
| CASP8 | TGFB2-AS1 | 0.57174 | 3.04E-47 | postive |
| NOD1 | TGFB2-AS1 | 0.543726 | 4.99E-42 | postive |
| GSDMB | FAM13A-AS1 | 0.705815 | 6.12E-81 | postive |
| CASP8 | AC015922.2 | 0.539854 | 2.40E-41 | postive |
| CASP1 | AC008750.1 | 0.52015 | 5.26E-38 | postive |
| IL18 | AC008750.1 | 0.55196 | 1.64E-43 | postive |
| NLRC4 | AC008750.1 | 0.534829 | 1.79E-40 | postive |
| GSDMB | AC002059.1 | 0.519288 | 7.28E-38 | postive |
| CASP1 | AC009878.1 | -0.5106 | 1.83E-36 | negative |
| NLRP2 | AL162311.1 | 0.510091 | 2.21E-36 | postive |
| SCAF11 | AL162311.1 | -0.50521 | 1.29E-35 | negative |
| NLRP2 | PART1 | 0.575888 | 4.65E-48 | postive |
| GSDMD | AC015908.2 | 0.532306 | 4.85E-40 | postive |
| GSDMB | AL158151.4 | 0.534009 | 2.48E-40 | postive |
| GSDMB | AC073655.2 | 0.665934 | 4.50E-69 | postive |
| PRKACA | BX322234.1 | 0.548566 | 6.79E-43 | postive |
| CASP1 | AC122707.1 | -0.54133 | 1.32E-41 | negative |
| CASP4 | AC122707.1 | -0.53175 | 6.03E-40 | negative |
| CASP6 | AC122707.1 | -0.50736 | 5.96E-36 | negative |
| CASP8 | AC122707.1 | -0.54895 | 5.79E-43 | negative |
| NOD1 | AC122707.1 | -0.58429 | 9.54E-50 | negative |
| GSDMB | LINC00926 | 0.666762 | 2.66E-69 | postive |
| NLRP2 | LINC01007 | 0.58997 | 6.48E-51 | postive |
| CASP1 | AC012213.1 | -0.60751 | 1.13E-54 | negative |
| CASP4 | AC012213.1 | -0.51145 | 1.34E-36 | negative |
| CASP6 | AC012213.1 | -0.59649 | 2.76E-52 | negative |
| CASP8 | AC012213.1 | -0.54945 | 4.70E-43 | negative |
| GSDMD | AC012213.1 | -0.55915 | 7.72E-45 | negative |
| IL18 | AC012213.1 | -0.56427 | 8.37E-46 | negative |
| NLRP3 | AC012213.1 | -0.50031 | 7.41E-35 | negative |
| NOD1 | AC012213.1 | -0.55318 | 9.84E-44 | negative |
| PYCARD | AC012213.1 | -0.57529 | 6.11E-48 | negative |
| GSDMB | AL121890.5 | 0.541062 | 1.47E-41 | postive |
| PJVK | AL121890.5 | 0.533871 | 2.62E-40 | postive |
| NLRP2 | AP001972.3 | 0.526515 | 4.63E-39 | postive |
| GSDMB | AL117209.1 | 0.507838 | 5.01E-36 | postive |
| CASP1 | AC010247.2 | 0.621212 | 8.84E-58 | postive |
| CASP4 | AC010247.2 | 0.506367 | 8.53E-36 | postive |
| IL18 | AC010247.2 | 0.629786 | 8.40E-60 | postive |
| NLRC4 | AC010247.2 | 0.63331 | 1.19E-60 | postive |
| NLRP3 | AC010247.2 | 0.582207 | 2.53E-49 | postive |
| PYCARD | AC010247.2 | 0.697416 | 2.81E-78 | postive |
| CASP3 | LINC01579 | 0.533864 | 2.63E-40 | postive |
| NLRP2 | AC003684.1 | 0.503417 | 2.46E-35 | postive |
| CASP4 | SLC25A21-AS1 | -0.52031 | 4.94E-38 | negative |
| CASP6 | SLC25A21-AS1 | -0.52166 | 2.97E-38 | negative |
| CASP8 | SLC25A21-AS1 | -0.58186 | 2.97E-49 | negative |
| GSDMD | SLC25A21-AS1 | -0.50943 | 2.81E-36 | negative |
| PRKACA | SLC25A21-AS1 | -0.57277 | 1.91E-47 | negative |
| CASP8 | AL359091.1 | -0.50203 | 4.03E-35 | negative |
| PJVK | AL359091.1 | 0.546741 | 1.45E-42 | postive |
| CASP8 | AC016876.1 | -0.54117 | 1.41E-41 | negative |
| CASP6 | AC093726.2 | 0.549647 | 4.33E-43 | postive |
| GSDMD | AC093726.2 | 0.637772 | 9.61E-62 | postive |
| CASP4 | AL121956.4 | -0.50324 | 2.62E-35 | negative |
| GSDMB | AC010883.1 | 0.581908 | 2.91E-49 | postive |
| PJVK | AC010883.1 | 0.550855 | 2.61E-43 | postive |
| CASP9 | BX293535.1 | 0.534733 | 1.86E-40 | postive |
| PRKACA | AL391834.2 | -0.60374 | 7.58E-54 | negative |
| CASP1 | BCDIN3D-AS1 | -0.53783 | 5.42E-41 | negative |
| CASP6 | BCDIN3D-AS1 | -0.52034 | 4.89E-38 | negative |
| GSDMD | BCDIN3D-AS1 | -0.5584 | 1.07E-44 | negative |
| IL18 | BCDIN3D-AS1 | -0.52132 | 3.37E-38 | negative |
| PYCARD | BCDIN3D-AS1 | -0.55795 | 1.29E-44 | negative |
| GSDMB | AL449403.1 | 0.53942 | 2.86E-41 | postive |
| GSDMD | SNHG18 | 0.627612 | 2.77E-59 | postive |
| NLRP3 | GNG12-AS1 | 0.508115 | 4.53E-36 | postive |
| NOD1 | GNG12-AS1 | 0.543221 | 6.13E-42 | postive |
| CASP9 | AL645608.6 | 0.579956 | 7.20E-49 | postive |
| NOD1 | DLGAP1-AS2 | 0.504335 | 1.77E-35 | postive |
| GSDMB | IGBP1-AS1 | 0.546745 | 1.44E-42 | postive |
| CASP1 | AC002091.1 | 0.66245 | 4.01E-68 | postive |
| CASP4 | AC002091.1 | 0.561317 | 3.03E-45 | postive |
| CASP8 | AC002091.1 | 0.589755 | 7.18E-51 | postive |
| IL18 | AC002091.1 | 0.750765 | 5.78E-97 | postive |
| NLRC4 | AC002091.1 | 0.681011 | 2.45E-73 | postive |
| NLRP3 | AC002091.1 | 0.658613 | 4.31E-67 | postive |
| PYCARD | AC002091.1 | 0.644557 | 1.94E-63 | postive |
| PYCARD | AC092809.2 | 0.510632 | 1.81E-36 | postive |
| TIRAP | AC004771.3 | 0.528442 | 2.20E-39 | postive |
| CASP6 | PHEX-AS1 | 0.506198 | 9.06E-36 | postive |
| GSDMB | AL138921.1 | 0.519458 | 6.83E-38 | postive |
| CASP6 | AGAP2-AS1 | 0.540286 | 2.02E-41 | postive |
| GSDMD | AGAP2-AS1 | 0.569557 | 8.08E-47 | postive |
| CASP1 | AC145098.1 | 0.666615 | 2.92E-69 | postive |
| CASP4 | AC145098.1 | 0.679736 | 5.75E-73 | postive |
| CASP8 | AC145098.1 | 0.636442 | 2.04E-61 | postive |
| IL18 | AC145098.1 | 0.614012 | 3.95E-56 | postive |
| NLRC4 | AC145098.1 | 0.67608 | 6.48E-72 | postive |
| NOD1 | AC145098.1 | 0.590787 | 4.38E-51 | postive |
| PYCARD | AC145098.1 | 0.540886 | 1.58E-41 | postive |
| GSDMB | AC027682.4 | 0.675546 | 9.21E-72 | postive |
| GSDMB | LENG8-AS1 | 0.580544 | 5.48E-49 | postive |
| PLCG1 | LENG8-AS1 | 0.519891 | 5.80E-38 | postive |
| GSDMB | AL022328.1 | 0.585919 | 4.44E-50 | postive |
| GSDMD | NEAT1 | 0.587049 | 2.60E-50 | postive |
| IL18 | NEAT1 | 0.536212 | 1.03E-40 | postive |
| GSDMB | AL162741.1 | 0.615091 | 2.25E-56 | postive |
| CASP1 | NFIA-AS2 | 0.554505 | 5.62E-44 | postive |
| IL18 | NFIA-AS2 | 0.507264 | 6.17E-36 | postive |
| NLRP3 | NFIA-AS2 | 0.541701 | 1.14E-41 | postive |
| PYCARD | NFIA-AS2 | 0.54636 | 1.69E-42 | postive |
| GSDMB | AC120053.1 | 0.666408 | 3.33E-69 | postive |
| PJVK | AC120053.1 | 0.522699 | 2.00E-38 | postive |
| CASP1 | AC079015.1 | 0.505597 | 1.13E-35 | postive |
| IL18 | AC079015.1 | 0.641315 | 1.27E-62 | postive |
| NLRC4 | AC079015.1 | 0.503875 | 2.09E-35 | postive |
| NLRP3 | AC079015.1 | 0.657707 | 7.52E-67 | postive |
| PYCARD | AC079015.1 | 0.705091 | 1.05E-80 | postive |
| GSDMB | AC092143.3 | 0.560717 | 3.93E-45 | postive |
| GSDMB | AF038458.2 | 0.61643 | 1.12E-56 | postive |
| NLRP2 | AC010931.2 | 0.638252 | 7.31E-62 | postive |
| GSDMD | TRAPPC12-AS1 | -0.50036 | 7.30E-35 | negative |
| CASP1 | AL162426.1 | -0.60661 | 1.77E-54 | negative |
| CASP4 | AL162426.1 | -0.55489 | 4.77E-44 | negative |
| CASP6 | AL162426.1 | -0.56898 | 1.04E-46 | negative |
| CASP8 | AL162426.1 | -0.50496 | 1.42E-35 | negative |
| GSDMD | AL162426.1 | -0.54865 | 6.56E-43 | negative |
| IL18 | AL162426.1 | -0.57747 | 2.26E-48 | negative |
| PYCARD | AL162426.1 | -0.58907 | 9.96E-51 | negative |
| GSDMD | BX537318.1 | 0.536082 | 1.09E-40 | postive |
| GSDMD | AL121894.2 | 0.506743 | 7.44E-36 | postive |
| GSDMB | AL133325.3 | 0.577331 | 2.41E-48 | postive |
| PRKACA | ZFAS1 | -0.51345 | 6.43E-37 | negative |
| GSDMB | AC092119.2 | 0.681741 | 1.50E-73 | postive |
| PJVK | AC092119.2 | 0.528144 | 2.47E-39 | postive |
| CASP1 | AC108134.3 | 0.544214 | 4.08E-42 | postive |
| CASP4 | AC108134.3 | 0.60982 | 3.45E-55 | postive |
| GSDMD | AC108134.3 | 0.557558 | 1.53E-44 | postive |
| CASP1 | PSMB8-AS1 | 0.695706 | 9.53E-78 | postive |
| CASP4 | PSMB8-AS1 | 0.683025 | 6.32E-74 | postive |
| CASP6 | PSMB8-AS1 | 0.576695 | 3.22E-48 | postive |
| CASP8 | PSMB8-AS1 | 0.568174 | 1.50E-46 | postive |
| GSDMD | PSMB8-AS1 | 0.624007 | 1.97E-58 | postive |
| IL18 | PSMB8-AS1 | 0.596897 | 2.26E-52 | postive |
| NLRC4 | PSMB8-AS1 | 0.524736 | 9.18E-39 | postive |
| PYCARD | PSMB8-AS1 | 0.59026 | 5.64E-51 | postive |
| NOD1 | AC004158.1 | -0.51271 | 8.45E-37 | negative |
| GSDMD | AC018450.1 | 0.501869 | 4.27E-35 | postive |
| GSDMB | ZNF436-AS1 | 0.541528 | 1.22E-41 | postive |
| CASP6 | AP000721.2 | -0.54437 | 3.83E-42 | negative |
| GSDMD | AP000721.2 | -0.58046 | 5.69E-49 | negative |
| TIRAP | AP000721.2 | 0.539892 | 2.36E-41 | postive |
| PJVK | LINC00653 | 0.51518 | 3.38E-37 | postive |
| TIRAP | AC026471.1 | 0.538736 | 3.77E-41 | postive |
| CASP1 | NECTIN3-AS1 | -0.55867 | 9.50E-45 | negative |
| CASP4 | NECTIN3-AS1 | -0.53437 | 2.15E-40 | negative |
| CASP6 | NECTIN3-AS1 | -0.54532 | 2.59E-42 | negative |
| CASP8 | NECTIN3-AS1 | -0.51087 | 1.66E-36 | negative |
| NOD1 | NECTIN3-AS1 | -0.50634 | 8.62E-36 | negative |
| SCAF11 | CRIM1-DT | -0.53471 | 1.88E-40 | negative |
| NLRP2 | HTR5A-AS1 | 0.594833 | 6.19E-52 | postive |
| NLRP2 | AC134312.4 | 0.547016 | 1.29E-42 | postive |
| GSDMD | LINC00630 | -0.59803 | 1.30E-52 | negative |
| PYCARD | LINC00630 | -0.60989 | 3.34E-55 | negative |
| SCAF11 | LINC00630 | 0.501862 | 4.28E-35 | postive |
| GSDMB | AL645608.1 | 0.665988 | 4.35E-69 | postive |
| CASP8 | AP001469.3 | -0.52485 | 8.78E-39 | negative |
| PRKACA | AP001469.3 | -0.55537 | 3.89E-44 | negative |
| CASP6 | AC011450.1 | 0.565093 | 5.83E-46 | postive |
| GSDMB | AP006621.2 | 0.509137 | 3.12E-36 | postive |
| CASP4 | AC062021.1 | -0.51506 | 3.54E-37 | negative |
| CASP6 | AC062021.1 | -0.54236 | 8.70E-42 | negative |
| CASP8 | AC062021.1 | -0.57078 | 4.68E-47 | negative |
| GSDMB | AC127024.4 | 0.593995 | 9.31E-52 | postive |
| NLRP2 | HCG11 | 0.501844 | 4.31E-35 | postive |
| GSDMD | AC006449.5 | -0.53118 | 7.55E-40 | negative |
| IL18 | AC006449.5 | -0.52896 | 1.80E-39 | negative |
| CASP1 | AC005696.4 | -0.61711 | 7.81E-57 | negative |
| CASP4 | AC005696.4 | -0.65888 | 3.65E-67 | negative |
| CASP6 | AC005696.4 | -0.60359 | 8.17E-54 | negative |
| CASP8 | AC005696.4 | -0.67228 | 7.77E-71 | negative |
| GSDMD | AC005696.4 | -0.50332 | 2.54E-35 | negative |
| IL18 | AC005696.4 | -0.54015 | 2.13E-41 | negative |
| NLRC4 | AC005696.4 | -0.51162 | 1.26E-36 | negative |
| NOD1 | AC005696.4 | -0.60516 | 3.69E-54 | negative |
| CASP1 | LBX2-AS1 | 0.600339 | 4.14E-53 | postive |
| CASP4 | LBX2-AS1 | 0.684097 | 3.05E-74 | postive |
| CASP6 | LBX2-AS1 | 0.550847 | 2.62E-43 | postive |
| CASP8 | LBX2-AS1 | 0.547098 | 1.25E-42 | postive |
| GSDMD | LBX2-AS1 | 0.594637 | 6.81E-52 | postive |
| NOD1 | LBX2-AS1 | 0.539067 | 3.30E-41 | postive |
| PYCARD | LBX2-AS1 | 0.505168 | 1.31E-35 | postive |
| GSDMB | AC007220.1 | 0.578441 | 1.45E-48 | postive |
| CASP1 | AL731567.1 | 0.605664 | 2.87E-54 | postive |
| CASP4 | AL731567.1 | 0.548672 | 6.50E-43 | postive |
| CASP8 | AL731567.1 | 0.542141 | 9.51E-42 | postive |
| IL18 | AL731567.1 | 0.590148 | 5.95E-51 | postive |
| NLRC4 | AL731567.1 | 0.527634 | 3.01E-39 | postive |
| NLRP3 | AL731567.1 | 0.523159 | 1.68E-38 | postive |
| NOD1 | AL731567.1 | 0.548681 | 6.47E-43 | postive |
| PYCARD | AL731567.1 | 0.552484 | 1.32E-43 | postive |
| NLRP2 | LINC01106 | 0.595561 | 4.34E-52 | postive |
| CASP1 | TRHDE-AS1 | -0.53806 | 4.93E-41 | negative |
| GSDMB | AL590666.2 | 0.519553 | 6.59E-38 | postive |
| PRKACA | AL590666.2 | -0.51184 | 1.16E-36 | negative |
| GSDMB | AC093788.1 | 0.595786 | 3.89E-52 | postive |
| PJVK | AL121655.1 | 0.644902 | 1.58E-63 | postive |
| GSDMB | LINC02604 | 0.573984 | 1.11E-47 | postive |
| GSDMB | AC008735.2 | 0.54713 | 1.23E-42 | postive |
| PLCG1 | AC008735.2 | 0.597571 | 1.62E-52 | postive |
| GSDMB | AL033384.2 | 0.531975 | 5.53E-40 | postive |
| PYCARD | NPTN-IT1 | -0.54256 | 8.01E-42 | negative |
| CASP4 | ADGRA1-AS1 | -0.5046 | 1.61E-35 | negative |
| CASP6 | ADGRA1-AS1 | -0.53854 | 4.08E-41 | negative |
| CASP8 | ADGRA1-AS1 | -0.52836 | 2.27E-39 | negative |
| SCAF11 | AL121583.1 | -0.54435 | 3.87E-42 | negative |
| GSDMB | TMEM147-AS1 | 0.586746 | 3.00E-50 | postive |
| PLCG1 | TMEM147-AS1 | 0.541721 | 1.13E-41 | postive |
| GSDMD | LINC01719 | -0.6049 | 4.22E-54 | negative |
| PYCARD | LINC01719 | -0.53187 | 5.76E-40 | negative |
| CASP4 | WAC-AS1 | -0.50749 | 5.68E-36 | negative |
| CASP8 | WAC-AS1 | -0.52387 | 1.28E-38 | negative |
| CASP1 | LINC01736 | 0.593834 | 1.01E-51 | postive |
| IL18 | LINC01736 | 0.734284 | 1.04E-90 | postive |
| NLRC4 | LINC01736 | 0.568323 | 1.40E-46 | postive |
| NLRP3 | LINC01736 | 0.676597 | 4.61E-72 | postive |
| PYCARD | LINC01736 | 0.718197 | 4.86E-85 | postive |
| SCAF11 | U62317.2 | -0.54061 | 1.77E-41 | negative |
| GSDMB | AC010245.2 | 0.621204 | 8.88E-58 | postive |
| GSDMB | AC092123.1 | 0.581189 | 4.06E-49 | postive |
| CASP8 | AL023806.1 | -0.50232 | 3.63E-35 | negative |
| PRKACA | AL023806.1 | -0.5118 | 1.18E-36 | negative |
| GSDMB | AP003419.3 | 0.515962 | 2.53E-37 | postive |
| PRKACA | LINC02609 | 0.538948 | 3.46E-41 | postive |
| GSDMB | AP002907.1 | 0.536798 | 8.19E-41 | postive |
| GSDMD | AC120114.1 | -0.53587 | 1.19E-40 | negative |
| TIRAP | AC120114.1 | 0.565342 | 5.23E-46 | postive |
| GPX4 | AC004112.1 | -0.51959 | 6.49E-38 | negative |
| PLCG1 | AC074138.1 | 0.514503 | 4.35E-37 | postive |
| CASP8 | RPARP-AS1 | -0.51231 | 9.79E-37 | negative |
| GSDMB | AL513320.1 | 0.669124 | 5.92E-70 | postive |
| PRKACA | LINC00884 | 0.510163 | 2.15E-36 | postive |
| GSDMB | AL161729.4 | 0.511399 | 1.37E-36 | postive |
| PRKACA | Z97989.1 | -0.51639 | 2.15E-37 | negative |
| GSDMB | AP002490.1 | 0.648485 | 1.93E-64 | postive |
| SCAF11 | SMG7-AS1 | 0.559865 | 5.68E-45 | postive |
| CASP1 | HCP5 | 0.689117 | 9.76E-76 | postive |
| CASP4 | HCP5 | 0.760364 | ###### | postive |
| CASP6 | HCP5 | 0.552227 | 1.47E-43 | postive |
| CASP8 | HCP5 | 0.650497 | 5.85E-65 | postive |
| GSDMD | HCP5 | 0.588574 | 1.26E-50 | postive |
| IL18 | HCP5 | 0.509141 | 3.12E-36 | postive |
| NLRC4 | HCP5 | 0.506116 | 9.33E-36 | postive |
| NOD1 | HCP5 | 0.554415 | 5.84E-44 | postive |
| CASP6 | AP001486.2 | -0.53805 | 4.96E-41 | negative |
| NOD1 | AC103923.1 | 0.500572 | 6.76E-35 | postive |
| CASP1 | RFPL1S | -0.55265 | 1.23E-43 | negative |
| NOD1 | RFPL1S | -0.55179 | 1.77E-43 | negative |
| PYCARD | RFPL1S | -0.52402 | 1.21E-38 | negative |
| CASP8 | SNAI3-AS1 | -0.50529 | 1.26E-35 | negative |
| NOD1 | SNAI3-AS1 | -0.5108 | 1.70E-36 | negative |
| SCAF11 | SNAI3-AS1 | -0.52522 | 7.62E-39 | negative |
| PLCG1 | AC109460.2 | 0.574587 | 8.41E-48 | postive |
| SCAF11 | AC109460.2 | 0.515759 | 2.73E-37 | postive |
| NLRP2 | MIR4500HG | 0.505613 | 1.12E-35 | postive |
| GSDMB | AC012615.6 | 0.54488 | 3.11E-42 | postive |
| PLCG1 | AC012615.6 | 0.55428 | 6.18E-44 | postive |
| CASP1 | SOX1-OT | -0.5069 | 7.02E-36 | negative |
| GSDMD | ZBTB20-AS4 | -0.57188 | 2.86E-47 | negative |
| TIRAP | ZBTB20-AS4 | 0.530716 | 9.06E-40 | postive |
| GSDMB | AL354836.1 | 0.521157 | 3.59E-38 | postive |
| GSDMB | AC012645.4 | 0.612948 | 6.87E-56 | postive |
| PJVK | AC012645.4 | 0.509798 | 2.46E-36 | postive |
| GSDMB | AC009283.1 | 0.671254 | 1.50E-70 | postive |
| PJVK | AC009283.1 | 0.591925 | 2.53E-51 | postive |
| CASP1 | AC099524.1 | 0.665427 | 6.20E-69 | postive |
| CASP4 | AC099524.1 | 0.510298 | 2.05E-36 | postive |
| CASP8 | AC099524.1 | 0.527731 | 2.90E-39 | postive |
| IL18 | AC099524.1 | 0.721925 | 2.56E-86 | postive |
| NLRC4 | AC099524.1 | 0.686782 | 4.88E-75 | postive |
| NLRP3 | AC099524.1 | 0.642246 | 7.40E-63 | postive |
| PYCARD | AC099524.1 | 0.741421 | 2.33E-93 | postive |
| GSDMB | AL391684.1 | 0.500262 | 7.55E-35 | postive |
| PJVK | AL391684.1 | 0.53369 | 2.81E-40 | postive |
| PLCG1 | AC008982.2 | 0.536258 | 1.02E-40 | postive |
| GSDMB | VPS9D1-AS1 | 0.508563 | 3.85E-36 | postive |
| GSDMD | AL133342.1 | 0.512802 | 8.16E-37 | postive |
| NLRP2 | AC062028.1 | 0.608611 | 6.42E-55 | postive |
| CASP8 | AC051619.4 | -0.51582 | 2.67E-37 | negative |
| PLCG1 | AC073842.2 | 0.603787 | 7.40E-54 | postive |
| CASP6 | AC009227.1 | -0.61153 | 1.43E-55 | negative |
| GSDMD | AC009227.1 | -0.62391 | 2.07E-58 | negative |
| GSDMB | AC010542.5 | 0.613252 | 5.87E-56 | postive |
| CASP8 | AC000068.2 | -0.55253 | 1.29E-43 | negative |
| PRKACA | AC000068.2 | -0.53878 | 3.70E-41 | negative |
| CASP1 | AL512353.1 | 0.673984 | 2.56E-71 | postive |
| CASP4 | AL512353.1 | 0.58954 | 7.96E-51 | postive |
| CASP6 | AL512353.1 | 0.514639 | 4.14E-37 | postive |
| CASP8 | AL512353.1 | 0.626234 | 5.88E-59 | postive |
| IL18 | AL512353.1 | 0.600781 | 3.32E-53 | postive |
| NLRC4 | AL512353.1 | 0.580731 | 5.03E-49 | postive |
| NLRP3 | AL512353.1 | 0.518449 | 9.98E-38 | postive |
| NOD1 | AL512353.1 | 0.632736 | 1.63E-60 | postive |
| PYCARD | AL512353.1 | 0.561242 | 3.13E-45 | postive |
| NLRP2 | LINC02192 | 0.566417 | 3.26E-46 | postive |
| GSDMB | AC012368.1 | 0.54093 | 1.55E-41 | postive |
| GSDMB | LINC02636 | 0.540444 | 1.89E-41 | postive |
| GSDMB | AC015961.2 | 0.697354 | 2.94E-78 | postive |
| CASP1 | AC022893.2 | -0.50745 | 5.76E-36 | negative |
| GSDMD | AC127070.2 | -0.5142 | 4.88E-37 | negative |
| NLRP2 | AC011995.2 | 0.521076 | 3.71E-38 | postive |
| PRKACA | LINC00237 | -0.50837 | 4.14E-36 | negative |
| GSDMB | AL008729.1 | 0.644451 | 2.06E-63 | postive |
| GSDMB | PSMA3-AS1 | 0.644466 | 2.04E-63 | postive |
| SCAF11 | AC108010.1 | 0.624574 | 1.45E-58 | postive |
| GSDMB | AC005519.1 | 0.578176 | 1.63E-48 | postive |
| CASP4 | AC023301.1 | -0.54827 | 7.67E-43 | negative |
| CASP6 | AC023301.1 | -0.52146 | 3.21E-38 | negative |
| CASP8 | AC023301.1 | -0.5073 | 6.09E-36 | negative |
| GSDMD | AC023301.1 | -0.56385 | 1.00E-45 | negative |
| NOD1 | AC023301.1 | -0.50205 | 4.01E-35 | negative |
| GSDMB | AL359881.3 | 0.521589 | 3.05E-38 | postive |
| GSDMB | AC092809.4 | 0.678292 | 1.50E-72 | postive |
| CASP1 | LINC01909 | 0.510547 | 1.87E-36 | postive |
| IL18 | LINC01909 | 0.547028 | 1.28E-42 | postive |
| PYCARD | LINC01909 | 0.679816 | 5.45E-73 | postive |
| CASP3 | PAXIP1-AS2 | 0.507266 | 6.16E-36 | postive |
| CASP6 | PAXIP1-AS2 | 0.529481 | 1.47E-39 | postive |
| CASP8 | PAXIP1-AS2 | 0.612304 | 9.60E-56 | postive |
| CASP1 | AC027307.2 | 0.554745 | 5.08E-44 | postive |
| CASP4 | AC027307.2 | 0.527107 | 3.69E-39 | postive |
| CASP6 | AC027307.2 | 0.60728 | 1.26E-54 | postive |
| GSDMD | AC027307.2 | 0.582918 | 1.82E-49 | postive |
| PYCARD | AC027307.2 | 0.510112 | 2.19E-36 | postive |
| GSDMD | RAB11B-AS1 | 0.516307 | 2.22E-37 | postive |
| CASP8 | AP000350.5 | -0.57332 | 1.49E-47 | negative |
| GSDMD | AL160313.1 | -0.50999 | 2.29E-36 | negative |
| GSDMC | AC021755.2 | 0.50482 | 1.49E-35 | postive |
| NLRP2 | PPP1R26-AS1 | 0.525651 | 6.46E-39 | postive |
| PRKACA | PPP1R26-AS1 | 0.509146 | 3.11E-36 | postive |
| CASP1 | SMCR5 | -0.52056 | 4.51E-38 | negative |
| GSDMD | LINC00863 | -0.54852 | 6.92E-43 | negative |
| SCAF11 | MAGI2-AS3 | 0.507962 | 4.79E-36 | postive |
| NLRP2 | AP003559.1 | 0.504895 | 1.45E-35 | postive |
| PYCARD | AL392172.1 | 0.51033 | 2.02E-36 | postive |
| GSDMD | AL158835.2 | -0.52401 | 1.21E-38 | negative |
| GSDMB | AL354760.1 | 0.534259 | 2.25E-40 | postive |
| GSDMB | AC027348.1 | 0.541354 | 1.31E-41 | postive |
| PJVK | AC027348.1 | 0.50015 | 7.85E-35 | postive |
| GSDMB | AC087741.1 | 0.771215 | ###### | postive |
| GSDMB | BCRP3 | 0.578385 | 1.48E-48 | postive |
| CASP1 | VIM-AS1 | 0.576432 | 3.63E-48 | postive |
| CASP4 | VIM-AS1 | 0.577328 | 2.41E-48 | postive |
| CASP6 | VIM-AS1 | 0.579619 | 8.41E-49 | postive |
| CASP8 | VIM-AS1 | 0.555477 | 3.72E-44 | postive |
| IL18 | VIM-AS1 | 0.50593 | 9.98E-36 | postive |
| NOD1 | VIM-AS1 | 0.592769 | 1.69E-51 | postive |
| PYCARD | VIM-AS1 | 0.511136 | 1.51E-36 | postive |
| IL18 | AL158071.3 | 0.544248 | 4.03E-42 | postive |
| PYCARD | AL158071.3 | 0.573743 | 1.23E-47 | postive |
| GSDMB | AC015813.1 | 0.765529 | ###### | postive |
| PLCG1 | AC015813.1 | 0.539658 | 2.60E-41 | postive |
| GSDMB | AL136295.6 | 0.513133 | 7.22E-37 | postive |
| GSDMB | RUSC1-AS1 | 0.600194 | 4.45E-53 | postive |
| CASP1 | AF106564.1 | -0.55006 | 3.64E-43 | negative |
| NOD1 | AF106564.1 | -0.50706 | 6.63E-36 | negative |
| CASP1 | HAR1A | -0.59017 | 5.89E-51 | negative |
| CASP4 | HAR1A | -0.56293 | 1.50E-45 | negative |
| CASP6 | HAR1A | -0.55128 | 2.19E-43 | negative |
| CASP8 | HAR1A | -0.56491 | 6.32E-46 | negative |
| IL18 | HAR1A | -0.50742 | 5.83E-36 | negative |
| NOD1 | HAR1A | -0.59635 | 2.96E-52 | negative |
| GSDMB | AL162511.1 | 0.572898 | 1.81E-47 | postive |
| SCAF11 | AC005562.1 | 0.511306 | 1.41E-36 | postive |
| GSDMD | AL731577.2 | -0.54279 | 7.31E-42 | negative |
| CASP1 | HAR1B | -0.54557 | 2.34E-42 | negative |
| CASP4 | HAR1B | -0.50729 | 6.10E-36 | negative |
| CASP8 | HAR1B | -0.5052 | 1.30E-35 | negative |
| NOD1 | HAR1B | -0.53628 | 1.01E-40 | negative |
| CASP1 | AC003070.1 | 0.520307 | 4.96E-38 | postive |
| IL18 | AC003070.1 | 0.542756 | 7.41E-42 | postive |
| NLRC4 | AC003070.1 | 0.503504 | 2.38E-35 | postive |
| GPX4 | YTHDF3-AS1 | 0.501967 | 4.12E-35 | postive |
| SCAF11 | AC104836.1 | -0.50142 | 5.02E-35 | negative |
| GSDMB | AP001029.2 | 0.608747 | 5.99E-55 | postive |
| GSDMB | SNHG1 | 0.57086 | 4.52E-47 | postive |
| PRKACA | SNHG1 | -0.55642 | 2.49E-44 | negative |
| GSDMD | AP001269.2 | 0.545605 | 2.31E-42 | postive |
| GSDMB | AL122125.1 | 0.589466 | 8.24E-51 | postive |
| PJVK | AL122125.1 | 0.544215 | 4.08E-42 | postive |
| CASP4 | EDRF1-DT | -0.50346 | 2.42E-35 | negative |
| GSDMD | EDRF1-DT | -0.55174 | 1.80E-43 | negative |
| SCAF11 | AC004492.1 | 0.591237 | 3.53E-51 | postive |
| GSDMB | AL117379.1 | 0.600477 | 3.86E-53 | postive |
| GSDMB | AC126118.1 | 0.670455 | 2.52E-70 | postive |
| PJVK | AC126118.1 | 0.554169 | 6.48E-44 | postive |
| GSDMB | AC114730.3 | 0.624672 | 1.37E-58 | postive |
| NLRP2 | AC007922.3 | 0.501169 | 5.48E-35 | postive |
| GSDMB | AC025287.3 | 0.594589 | 6.97E-52 | postive |
| CASP8 | AC012508.2 | -0.50451 | 1.66E-35 | negative |
| NOD1 | AC012508.2 | -0.50467 | 1.57E-35 | negative |
| GSDMB | AC010973.2 | 0.620745 | 1.13E-57 | postive |
| GSDMB | AC002553.1 | 0.579251 | 9.96E-49 | postive |
| CASP1 | AC092131.1 | 0.535253 | 1.51E-40 | postive |
| SCAF11 | AC092171.5 | -0.5487 | 6.42E-43 | negative |
| GSDMB | SCAT2 | 0.511666 | 1.24E-36 | postive |
| GSDMB | AC009148.1 | 0.559883 | 5.63E-45 | postive |
| PJVK | AC009148.1 | 0.518443 | 1.00E-37 | postive |
| CASP1 | AL137186.2 | 0.60118 | 2.72E-53 | postive |
| IL18 | AL137186.2 | 0.64158 | 1.09E-62 | postive |
| NLRC4 | AL137186.2 | 0.663279 | 2.39E-68 | postive |
| NLRP3 | AL137186.2 | 0.585592 | 5.18E-50 | postive |
| PYCARD | AL137186.2 | 0.749755 | 1.44E-96 | postive |
| NLRP3 | AL135960.1 | 0.560385 | 4.54E-45 | postive |
| TNF | AL135960.1 | 0.549959 | 3.80E-43 | postive |
| SCAF11 | FGD5-AS1 | 0.553346 | 9.17E-44 | postive |
| SCAF11 | AC015726.1 | -0.53936 | 2.94E-41 | negative |
| GSDMD | AC073389.1 | -0.50082 | 6.19E-35 | negative |
| GSDMB | MALAT1 | 0.596027 | 3.46E-52 | postive |
| TIRAP | AP000866.1 | 0.503229 | 2.63E-35 | postive |
| GSDMB | AC127024.5 | 0.588673 | 1.20E-50 | postive |
| PJVK | AC024075.2 | 0.515629 | 2.86E-37 | postive |
| SCAF11 | AC006538.1 | -0.50699 | 6.82E-36 | negative |
| GSDMB | AL355987.4 | 0.712083 | 5.47E-83 | postive |
| GSDMB | AC010761.2 | 0.525629 | 6.52E-39 | postive |
| IL18 | AC012150.1 | 0.571728 | 3.06E-47 | postive |
| NLRP3 | AC012150.1 | 0.628336 | 1.87E-59 | postive |
| GPX4 | AC040169.1 | 0.581801 | 3.06E-49 | postive |
| CASP1 | DGCR5 | -0.51481 | 3.88E-37 | negative |
| CASP4 | DGCR5 | -0.5054 | 1.21E-35 | negative |
| NOD1 | DGCR5 | -0.55857 | 9.92E-45 | negative |
| IL18 | LINC01374 | 0.572687 | 1.99E-47 | postive |
| NLRP3 | LINC01374 | 0.65184 | 2.62E-65 | postive |
| PYCARD | LINC01374 | 0.61192 | 1.17E-55 | postive |
| CASP9 | LINC02593 | 0.553592 | 8.27E-44 | postive |
| CASP1 | INHBA-AS1 | -0.5101 | 2.20E-36 | negative |
| NLRP2 | LINC01123 | 0.577061 | 2.72E-48 | postive |
| GSDMB | LINC02094 | 0.503118 | 2.74E-35 | postive |
| GSDMD | LINC01137 | 0.516313 | 2.22E-37 | postive |
| GSDMD | AC009974.1 | 0.528137 | 2.47E-39 | postive |
| IL18 | AC243960.3 | 0.519532 | 6.64E-38 | postive |
| IL1B | AC243960.3 | 0.605542 | 3.05E-54 | postive |
| NLRP3 | AC243960.3 | 0.697346 | 2.95E-78 | postive |
| PYCARD | AC243960.3 | 0.51675 | 1.89E-37 | postive |
| TNF | AC243960.3 | 0.65679 | 1.32E-66 | postive |
| GSDMB | AL021368.3 | 0.619921 | 1.76E-57 | postive |
| CASP1 | AC243960.1 | 0.694148 | 2.88E-77 | postive |
| CASP4 | AC243960.1 | 0.519673 | 6.30E-38 | postive |
| CASP8 | AC243960.1 | 0.532483 | 4.53E-40 | postive |
| IL18 | AC243960.1 | 0.73505 | 5.45E-91 | postive |
| NLRC4 | AC243960.1 | 0.710702 | 1.56E-82 | postive |
| NLRP3 | AC243960.1 | 0.69838 | 1.41E-78 | postive |
| NOD1 | AC243960.1 | 0.529864 | 1.26E-39 | postive |
| PYCARD | AC243960.1 | 0.753643 | 4.16E-98 | postive |
| GSDMB | AL049795.1 | 0.533075 | 3.59E-40 | postive |
| CASP1 | AL161785.1 | 0.748522 | 4.38E-96 | postive |
| CASP4 | AL161785.1 | 0.578112 | 1.68E-48 | postive |
| CASP8 | AL161785.1 | 0.53339 | 3.17E-40 | postive |
| IL18 | AL161785.1 | 0.819654 | ###### | postive |
| NLRC4 | AL161785.1 | 0.764854 | ###### | postive |
| NLRP3 | AL161785.1 | 0.668509 | 8.76E-70 | postive |
| NOD1 | AL161785.1 | 0.538309 | 4.47E-41 | postive |
| PYCARD | AL161785.1 | 0.844464 | ###### | postive |
| PYCARD | SENCR | 0.525948 | 5.76E-39 | postive |
| SCAF11 | PRKAG2-AS1 | -0.52212 | 2.49E-38 | negative |
| PRKACA | C21orf62-AS1 | -0.5031 | 2.75E-35 | negative |
| NLRP2 | C22orf24 | 0.55055 | 2.97E-43 | postive |
| GSDMB | RBFADN | 0.528923 | 1.82E-39 | postive |
| GSDMB | Z83843.1 | 0.517299 | 1.54E-37 | postive |
| CASP4 | AC074286.1 | -0.51101 | 1.58E-36 | negative |
| CASP6 | AC074286.1 | -0.6016 | 2.21E-53 | negative |
| CASP8 | AC074286.1 | -0.60503 | 3.95E-54 | negative |
| GSDMD | AC074286.1 | -0.58954 | 7.96E-51 | negative |
| PRKACA | AC074286.1 | -0.50637 | 8.51E-36 | negative |
| CASP1 | AL133163.3 | -0.5342 | 2.30E-40 | negative |
| IL18 | AL133163.3 | -0.51087 | 1.66E-36 | negative |
| NOD1 | AL133163.3 | -0.51085 | 1.67E-36 | negative |
| PRKACA | AL391834.1 | -0.50779 | 5.11E-36 | negative |
| NOD1 | AP001267.2 | -0.50421 | 1.85E-35 | negative |
| GSDMB | AC022167.2 | 0.524198 | 1.13E-38 | postive |
| GSDMB | CAPN10-DT | 0.611254 | 1.65E-55 | postive |
| GSDMB | AL031186.1 | 0.622731 | 3.91E-58 | postive |
| NLRP2 | AC004817.4 | 0.525287 | 7.43E-39 | postive |
| GSDMB | AC132872.3 | 0.583941 | 1.13E-49 | postive |
| CASP4 | CYTOR | 0.620286 | 1.45E-57 | postive |
| CASP6 | CYTOR | 0.505552 | 1.14E-35 | postive |
| GSDMD | CYTOR | 0.509723 | 2.52E-36 | postive |
| GSDMB | AC004253.1 | 0.661529 | 7.11E-68 | postive |
| CASP1 | RUNDC3A-AS1 | -0.70496 | 1.16E-80 | negative |
| CASP4 | RUNDC3A-AS1 | -0.60118 | 2.73E-53 | negative |
| CASP6 | RUNDC3A-AS1 | -0.60149 | 2.33E-53 | negative |
| CASP8 | RUNDC3A-AS1 | -0.62775 | 2.57E-59 | negative |
| GSDMD | RUNDC3A-AS1 | -0.52333 | 1.57E-38 | negative |
| IL18 | RUNDC3A-AS1 | -0.62834 | 1.86E-59 | negative |
| NLRC4 | RUNDC3A-AS1 | -0.54953 | 4.54E-43 | negative |
| NLRP3 | RUNDC3A-AS1 | -0.55024 | 3.37E-43 | negative |
| NOD1 | RUNDC3A-AS1 | -0.62433 | 1.65E-58 | negative |
| PYCARD | RUNDC3A-AS1 | -0.59353 | 1.16E-51 | negative |
| GPX4 | AC234775.3 | -0.53492 | 1.73E-40 | negative |
| CASP8 | AL662844.4 | -0.53287 | 3.88E-40 | negative |
| IL18 | AC008759.3 | 0.531569 | 6.48E-40 | postive |
| NLRP3 | AC008759.3 | 0.594722 | 6.54E-52 | postive |
| CASP8 | AL354892.2 | -0.50734 | 6.00E-36 | negative |
| PRKACA | AL354892.2 | -0.54442 | 3.75E-42 | negative |
| PRKACA | CPNE8-AS1 | 0.566396 | 3.29E-46 | postive |
| GPX4 | RAP2C-AS1 | -0.5051 | 1.35E-35 | negative |
| SCAF11 | RAP2C-AS1 | 0.568316 | 1.40E-46 | postive |
| CASP8 | AC004233.1 | -0.50935 | 2.89E-36 | negative |
| GSDMB | AC011815.2 | 0.520493 | 4.62E-38 | postive |
| NLRP2 | LINC02668 | 0.567393 | 2.11E-46 | postive |
| NLRP2 | THSD4-AS1 | 0.587124 | 2.51E-50 | postive |
| GPX4 | AC133552.5 | 0.521208 | 3.52E-38 | postive |
| PJVK | AC133552.5 | 0.500502 | 6.93E-35 | postive |
| PYCARD | AC008764.2 | -0.50475 | 1.52E-35 | negative |
| CASP1 | LINC02559 | -0.54538 | 2.54E-42 | negative |
| CASP8 | LINC02559 | -0.53448 | 2.05E-40 | negative |
| NOD1 | LINC02559 | -0.50343 | 2.45E-35 | negative |
| GSDMB | AL136295.2 | 0.574757 | 7.79E-48 | postive |
| GSDMB | AC245884.1 | 0.596677 | 2.52E-52 | postive |
| GSDMB | AC011498.6 | 0.515259 | 3.29E-37 | postive |
| PYCARD | AL450263.1 | -0.53462 | 1.94E-40 | negative |
| SCAF11 | AL450263.1 | 0.510237 | 2.09E-36 | postive |
| IL18 | LINC01094 | 0.519399 | 6.98E-38 | postive |
| NLRP3 | LINC01094 | 0.576194 | 4.05E-48 | postive |
| GSDMB | AC016876.2 | 0.595238 | 5.09E-52 | postive |
| CASP1 | HLA-DQB1-AS1 | 0.573909 | 1.14E-47 | postive |
| CASP4 | HLA-DQB1-AS1 | 0.564062 | 9.16E-46 | postive |
| CASP8 | HLA-DQB1-AS1 | 0.523277 | 1.60E-38 | postive |
| IL18 | HLA-DQB1-AS1 | 0.527433 | 3.25E-39 | postive |
| CASP1 | TOB1-AS1 | -0.52798 | 2.63E-39 | negative |
| CASP4 | TOB1-AS1 | -0.52195 | 2.66E-38 | negative |
| CASP6 | TOB1-AS1 | -0.50626 | 8.86E-36 | negative |
| CASP8 | TOB1-AS1 | -0.65035 | 6.40E-65 | negative |
| NOD1 | TOB1-AS1 | -0.54404 | 4.38E-42 | negative |
| CASP4 | AC110285.1 | -0.51706 | 1.68E-37 | negative |
| CASP6 | AC110285.1 | -0.53054 | 9.69E-40 | negative |
| CASP8 | AC110285.1 | -0.52116 | 3.58E-38 | negative |
| NLRP2 | LINC00460 | 0.603108 | 1.04E-53 | postive |
| PRKACA | LINC00460 | 0.506837 | 7.19E-36 | postive |
| CASP6 | LINC00601 | 0.500661 | 6.55E-35 | postive |
| CASP8 | LINC00601 | 0.579023 | 1.11E-48 | postive |
| GSDMB | AC073487.1 | 0.59661 | 2.60E-52 | postive |
| GSDMB | AC017083.1 | 0.554159 | 6.51E-44 | postive |
| PJVK | AC017083.1 | 0.518329 | 1.04E-37 | postive |
| CASP9 | AC098587.1 | 0.533607 | 2.91E-40 | postive |
| GSDMB | AC018635.2 | 0.569241 | 9.31E-47 | postive |
| NLRP2 | AL049775.1 | 0.606733 | 1.67E-54 | postive |
| CASP1 | AC016717.2 | -0.56179 | 2.47E-45 | negative |
| CASP4 | AC016717.2 | -0.51997 | 5.64E-38 | negative |
| CASP6 | AC016717.2 | -0.52939 | 1.52E-39 | negative |
| GSDMD | AC016717.2 | -0.5566 | 2.30E-44 | negative |
| IL18 | AC016717.2 | -0.51995 | 5.68E-38 | negative |
| NOD1 | AC016717.2 | -0.55609 | 2.86E-44 | negative |
| PYCARD | AC016717.2 | -0.54371 | 5.01E-42 | negative |
| GSDMB | LINC01089 | 0.689151 | 9.53E-76 | postive |
| PJVK | LINC01089 | 0.525204 | 7.67E-39 | postive |
| SCAF11 | SEMA6A-AS1 | 0.550892 | 2.57E-43 | postive |
| SCAF11 | AC005726.3 | -0.5071 | 6.54E-36 | negative |
| GSDMB | PRKCZ-AS1 | 0.526817 | 4.12E-39 | postive |
| CASP1 | AL135999.2 | -0.50124 | 5.33E-35 | negative |
| CASP4 | AL135999.2 | -0.5459 | 2.04E-42 | negative |
| CASP6 | AL135999.2 | -0.50985 | 2.41E-36 | negative |
| GSDMB | SSBP3-AS1 | 0.6661 | 4.05E-69 | postive |
| CASP6 | AC002456.1 | 0.506475 | 8.20E-36 | postive |
| GSDMD | AC002456.1 | 0.501855 | 4.29E-35 | postive |
| GSDMB | AC110285.3 | 0.515576 | 2.92E-37 | postive |
| CASP1 | ITGB2-AS1 | 0.617563 | 6.14E-57 | postive |
| CASP4 | ITGB2-AS1 | 0.501578 | 4.74E-35 | postive |
| CASP8 | ITGB2-AS1 | 0.519824 | 5.95E-38 | postive |
| GSDMD | ITGB2-AS1 | 0.589248 | 9.14E-51 | postive |
| IL18 | ITGB2-AS1 | 0.662666 | 3.50E-68 | postive |
| NLRC4 | ITGB2-AS1 | 0.52901 | 1.76E-39 | postive |
| NLRP3 | ITGB2-AS1 | 0.54622 | 1.79E-42 | postive |
| PYCARD | ITGB2-AS1 | 0.699022 | 8.85E-79 | postive |
| GSDMB | AC005670.1 | 0.516885 | 1.79E-37 | postive |
| PJVK | AC005670.1 | 0.505242 | 1.28E-35 | postive |
| CASP8 | AC024560.3 | -0.54514 | 2.80E-42 | negative |
| NLRP2 | AP003355.2 | 0.556007 | 2.97E-44 | postive |
| GSDMB | AL135999.1 | 0.545534 | 2.38E-42 | postive |
| PLCG1 | AL135999.1 | 0.503568 | 2.33E-35 | postive |
| PYCARD | LINC02175 | -0.51282 | 8.12E-37 | negative |
| GSDMB | AL365330.1 | 0.64568 | 1.01E-63 | postive |
| PLCG1 | AL365330.1 | 0.537341 | 6.59E-41 | postive |
| GSDMB | LINC00158 | 0.595281 | 4.98E-52 | postive |
| GSDMB | AC011462.5 | 0.654715 | 4.64E-66 | postive |
| GSDMB | FSIP2-AS1 | 0.509255 | 2.99E-36 | postive |
| PRKACA | LINC00900 | 0.500786 | 6.27E-35 | postive |
| NLRP2 | LINC01197 | 0.540205 | 2.08E-41 | postive |
| NLRP2 | LINC02217 | 0.570494 | 5.32E-47 | postive |
| SCAF11 | AC025171.2 | 0.576677 | 3.25E-48 | postive |
| CASP1 | AL354919.2 | 0.608621 | 6.38E-55 | postive |
| CASP4 | AL354919.2 | 0.626247 | 5.84E-59 | postive |
| CASP6 | AL354919.2 | 0.5034 | 2.47E-35 | postive |
| CASP8 | AL354919.2 | 0.530153 | 1.13E-39 | postive |
| NLRC4 | AL354919.2 | 0.56699 | 2.53E-46 | postive |
| NOD1 | AL354919.2 | 0.541641 | 1.17E-41 | postive |
| CASP8 | OSER1-DT | -0.50771 | 5.25E-36 | negative |
| CASP4 | AC083862.2 | 0.575221 | 6.31E-48 | postive |
| CASP1 | AL031658.2 | -0.50784 | 5.00E-36 | negative |
| NLRP3 | AL031658.2 | -0.52536 | 7.24E-39 | negative |
| NOD1 | AL031658.2 | -0.53748 | 6.23E-41 | negative |
| CASP1 | AC090559.1 | 0.642167 | 7.75E-63 | postive |
| CASP4 | AC090559.1 | 0.510066 | 2.23E-36 | postive |
| CASP8 | AC090559.1 | 0.570715 | 4.82E-47 | postive |
| IL18 | AC090559.1 | 0.680732 | 2.96E-73 | postive |
| NLRC4 | AC090559.1 | 0.663697 | 1.84E-68 | postive |
| NLRP3 | AC090559.1 | 0.763977 | ###### | postive |
| NOD1 | AC090559.1 | 0.513179 | 7.10E-37 | postive |
| PYCARD | AC090559.1 | 0.575991 | 4.44E-48 | postive |
| CASP1 | AP000439.3 | 0.509207 | 3.05E-36 | postive |
| NLRC4 | AP000439.3 | 0.576359 | 3.75E-48 | postive |
| NLRP3 | AP000439.3 | 0.508522 | 3.91E-36 | postive |
| PYCARD | AP000439.3 | 0.666291 | 3.59E-69 | postive |
| GSDMB | LINC01311 | 0.635191 | 4.13E-61 | postive |
| CASP4 | MIR9-3HG | -0.5229 | 1.85E-38 | negative |
| CASP6 | MIR9-3HG | -0.55817 | 1.18E-44 | negative |
| CASP8 | MIR9-3HG | -0.58032 | 6.07E-49 | negative |
| CASP9 | MIR9-3HG | 0.509117 | 3.15E-36 | postive |
| GSDMD | MIR9-3HG | -0.52588 | 5.91E-39 | negative |
| PRKACA | MIR9-3HG | -0.55706 | 1.89E-44 | negative |
| CASP4 | PVT1 | 0.509216 | 3.04E-36 | postive |
| CASP4 | TMEM254-AS1 | -0.55451 | 5.61E-44 | negative |
| CASP8 | TMEM254-AS1 | -0.50273 | 3.14E-35 | negative |
| GSDMD | ARPP21-AS1 | -0.51622 | 2.30E-37 | negative |
| PRKACA | ARPP21-AS1 | -0.50783 | 5.02E-36 | negative |
| CASP6 | LINC02587 | 0.522972 | 1.80E-38 | postive |
| GSDMD | LINC02587 | 0.551248 | 2.22E-43 | postive |
| GSDMB | AC110285.2 | 0.646113 | 7.81E-64 | postive |
| PJVK | AC110285.2 | 0.549339 | 4.92E-43 | postive |
| CASP1 | AC092720.1 | -0.52506 | 8.12E-39 | negative |
| IL18 | AC092720.1 | -0.50949 | 2.74E-36 | negative |
| PYCARD | AC092720.1 | -0.54802 | 8.52E-43 | negative |
| PLCG1 | AC015849.3 | 0.517538 | 1.40E-37 | postive |
| CASP6 | LINC01270 | 0.50762 | 5.42E-36 | postive |
| CASP8 | LINC01270 | 0.505481 | 1.17E-35 | postive |
| NLRP2 | AL022323.1 | 0.511075 | 1.54E-36 | postive |
| GSDMB | AC021205.3 | 0.547868 | 9.07E-43 | postive |
| GSDMB | AL159169.2 | 0.64734 | 3.80E-64 | postive |
| CASP1 | AL589765.1 | -0.51035 | 2.01E-36 | negative |
| PYCARD | AL589765.1 | -0.50056 | 6.80E-35 | negative |
| NLRP2 | FAM87A | 0.533609 | 2.90E-40 | postive |
| GSDMB | AC005253.1 | 0.531317 | 7.16E-40 | postive |
| PLCG1 | AC005253.1 | 0.534658 | 1.92E-40 | postive |
| CASP1 | MIR124-2HG | -0.64846 | 1.97E-64 | negative |
| CASP4 | MIR124-2HG | -0.61143 | 1.51E-55 | negative |
| CASP6 | MIR124-2HG | -0.56962 | 7.87E-47 | negative |
| CASP8 | MIR124-2HG | -0.614 | 3.97E-56 | negative |
| IL18 | MIR124-2HG | -0.59826 | 1.16E-52 | negative |
| NLRC4 | MIR124-2HG | -0.5085 | 3.93E-36 | negative |
| NOD1 | MIR124-2HG | -0.63411 | 7.59E-61 | negative |
| PYCARD | MIR124-2HG | -0.57523 | 6.28E-48 | negative |
| PYCARD | AC069549.1 | 0.563753 | 1.05E-45 | postive |
| CASP1 | VSTM2A-OT1 | -0.55414 | 6.57E-44 | negative |
| NOD1 | VSTM2A-OT1 | -0.51802 | 1.17E-37 | negative |
| PYCARD | VSTM2A-OT1 | -0.52842 | 2.22E-39 | negative |
| CASP8 | ASH1L-AS1 | -0.50392 | 2.05E-35 | negative |
| PJVK | AL158063.1 | 0.561659 | 2.61E-45 | postive |
| GSDMD | AC079089.1 | -0.58625 | 3.79E-50 | negative |
| NLRP2 | LINC01476 | 0.594791 | 6.32E-52 | postive |
| CASP1 | CARD8-AS1 | 0.769374 | ###### | postive |
| CASP4 | CARD8-AS1 | 0.79316 | ###### | postive |
| CASP6 | CARD8-AS1 | 0.561366 | 2.97E-45 | postive |
| CASP8 | CARD8-AS1 | 0.734787 | 6.80E-91 | postive |
| GSDMD | CARD8-AS1 | 0.568661 | 1.20E-46 | postive |
| IL18 | CARD8-AS1 | 0.687915 | 2.24E-75 | postive |
| NLRC4 | CARD8-AS1 | 0.704456 | 1.67E-80 | postive |
| NLRP3 | CARD8-AS1 | 0.516148 | 2.36E-37 | postive |
| NOD1 | CARD8-AS1 | 0.682691 | 7.92E-74 | postive |
| PYCARD | CARD8-AS1 | 0.628624 | 1.59E-59 | postive |
| CASP4 | ZFHX2-AS1 | -0.57794 | 1.82E-48 | negative |
| CASP6 | ZFHX2-AS1 | -0.5053 | 1.25E-35 | negative |
| CASP8 | ZFHX2-AS1 | -0.5653 | 5.31E-46 | negative |
| GSDMB | LINC02615 | 0.523684 | 1.37E-38 | postive |
| PJVK | LINC02615 | 0.51433 | 4.64E-37 | postive |
| PJVK | AC004982.1 | 0.567582 | 1.94E-46 | postive |
| GSDMD | LINC01176 | 0.512621 | 8.72E-37 | postive |
| PYCARD | LINC01176 | 0.586449 | 3.46E-50 | postive |
| GSDMB | AC117395.1 | 0.505528 | 1.15E-35 | postive |
| GSDMB | AC068620.2 | 0.508009 | 4.71E-36 | postive |
| PJVK | AC068620.2 | 0.564974 | 6.14E-46 | postive |
| GSDMB | AL021707.6 | 0.520668 | 4.32E-38 | postive |
| GSDMB | TTC28-AS1 | 0.514296 | 4.70E-37 | postive |
| PRKACA | TTC28-AS1 | -0.54466 | 3.40E-42 | negative |
| PRKACA | AC128687.2 | 0.519176 | 7.59E-38 | postive |
| CASP3 | AC025171.5 | 0.502552 | 3.35E-35 | postive |
| CASP6 | AC025171.5 | 0.580794 | 4.88E-49 | postive |
| CASP8 | AC025171.5 | 0.5162 | 2.31E-37 | postive |
| GSDMD | AC025171.5 | 0.600721 | 3.42E-53 | postive |
| CASP1 | CISTR | -0.52758 | 3.07E-39 | negative |
| GSDMD | AC020916.1 | 0.536204 | 1.04E-40 | postive |
| CASP9 | AL645608.2 | 0.568126 | 1.53E-46 | postive |
| GSDMB | SLC9A3-AS1 | 0.515473 | 3.03E-37 | postive |
| GSDMB | AC022558.3 | 0.521327 | 3.37E-38 | postive |
| NLRP3 | LINC01141 | 0.61578 | 1.57E-56 | postive |
| PYCARD | LINC01141 | 0.556411 | 2.50E-44 | postive |
| GSDMB | AP002812.2 | 0.595009 | 5.69E-52 | postive |
| GSDMB | AC008610.1 | 0.538842 | 3.61E-41 | postive |
| PJVK | AC008610.1 | 0.54996 | 3.80E-43 | postive |
| CASP3 | AL158212.3 | -0.51651 | 2.07E-37 | negative |
| CASP4 | AL158212.3 | -0.55941 | 6.91E-45 | negative |
| CASP6 | AL158212.3 | -0.61488 | 2.52E-56 | negative |
| GSDMD | AL158212.3 | -0.5552 | 4.19E-44 | negative |
| PYCARD | LINC01678 | 0.562409 | 1.89E-45 | postive |
| CASP6 | AC015908.3 | 0.566978 | 2.54E-46 | postive |
| CASP8 | AC015908.3 | 0.555269 | 4.06E-44 | postive |
| GSDMD | AC015908.3 | 0.631229 | 3.78E-60 | postive |
| GSDMB | AC002553.2 | 0.664423 | 1.17E-68 | postive |
| PYCARD | ZNF346-IT1 | -0.54482 | 3.18E-42 | negative |
| PYCARD | ACAP2-IT1 | -0.57853 | 1.39E-48 | negative |
| PJVK | AL118558.4 | 0.531447 | 6.80E-40 | postive |
| GSDMB | AC092171.4 | 0.657223 | 1.01E-66 | postive |
| PJVK | AC092171.4 | 0.549932 | 3.84E-43 | postive |
| NLRP2 | AC005865.2 | 0.512978 | 7.65E-37 | postive |
| CASP6 | HOXB-AS1 | 0.553394 | 8.99E-44 | postive |
| GSDMD | HOXB-AS1 | 0.530967 | 8.21E-40 | postive |
| CASP4 | AC009041.2 | -0.51262 | 8.71E-37 | negative |
| CASP6 | AC009041.2 | -0.50057 | 6.77E-35 | negative |
| CASP8 | AC009041.2 | -0.5815 | 3.52E-49 | negative |
| GSDMB | AC009041.2 | 0.515494 | 3.01E-37 | postive |
| PRKACA | AC009041.2 | -0.51191 | 1.13E-36 | negative |
| GSDMB | AC018638.6 | 0.577106 | 2.67E-48 | postive |
| CASP1 | LINC01586 | -0.59471 | 6.59E-52 | negative |
| IL18 | LINC01586 | -0.54868 | 6.49E-43 | negative |
| NOD1 | LINC01586 | -0.5761 | 4.22E-48 | negative |
| PYCARD | LINC01586 | -0.56419 | 8.66E-46 | negative |
| PRKACA | ALOX12-AS1 | -0.51194 | 1.12E-36 | negative |
| GSDMD | AF131215.5 | -0.51317 | 7.12E-37 | negative |
| GSDMB | ZNF32-AS1 | 0.571124 | 4.01E-47 | postive |
| CASP1 | AC004067.1 | 0.500635 | 6.62E-35 | postive |
| CASP4 | AC004067.1 | 0.581777 | 3.09E-49 | postive |
| CASP6 | AC004067.1 | 0.702817 | 5.59E-80 | postive |
| CASP8 | AC004067.1 | 0.593724 | 1.06E-51 | postive |
| GSDMD | AC004067.1 | 0.623266 | 2.94E-58 | postive |
| NLRP2 | CELF2-DT | 0.578285 | 1.55E-48 | postive |
| PLCG1 | PLCG1-AS1 | 0.539737 | 2.52E-41 | postive |
| NLRP2 | LINC02607 | 0.571036 | 4.17E-47 | postive |
| CASP1 | AC097468.2 | -0.61128 | 1.63E-55 | negative |
| CASP6 | AC097468.2 | -0.52481 | 8.92E-39 | negative |
| CASP8 | AC097468.2 | -0.51324 | 6.94E-37 | negative |
| IL18 | AC097468.2 | -0.58184 | 3.00E-49 | negative |
| NLRP3 | AC097468.2 | -0.53566 | 1.29E-40 | negative |
| NOD1 | AC097468.2 | -0.55831 | 1.11E-44 | negative |
| PYCARD | AC097468.2 | -0.57065 | 4.96E-47 | negative |
| GSDMB | CCDC18-AS1 | 0.64905 | 1.38E-64 | postive |
| GSDMB | AC009118.3 | 0.584366 | 9.22E-50 | postive |
| GSDMB | AC132192.2 | 0.514457 | 4.43E-37 | postive |
| PJVK | AC132192.2 | 0.562944 | 1.49E-45 | postive |
| GSDMD | LINC01521 | -0.65391 | 7.54E-66 | negative |
| GSDMD | AL031666.1 | 0.547346 | 1.13E-42 | postive |
