# Supplementary material for: Machine Learning-Based Integration Develops a Pyroptosis-Related lncRNA Model to Enhance the Predicted Value of Low-Grade Glioma Patients
Source: J Oncol. 2022 May 19;2022:8164756. doi: 10.1155/2022/8164756 (PMC9135526; doi:10.1155/2022/8164756)
Supplement: Supplementary Materials — Supplementary File Table S1: 33 pyroptosis-related genes from prior reviews. Supplementary File Table S2: patients' clinical characteristics from TCGA-LGG. Supplementary File Table S3: 4 pyroptosis-related DEGs from TCGA-LGG. Supplementary File Table S4: 859 pyroptosis-related lncRNAs. Supplementary File Table S5: 77 significant pyroptosis-related lncRNAs after univariate Cox analysis. [file 8164756.f1.zip › Table S5.docx]

**Table S4.** 77 significant pyroptosis-related lncRNAs after univariate Cox analysis

| gene | HR | HR.95L | HR.95H | pvalue |
| --- | --- | --- | --- | --- |
| C2orf27A | 0.584225 | 0.49764 | 0.685875 | 5.13E-11 |
| AC124312.2 | 0.723513 | 0.617668 | 0.847496 | 6.06E-05 |
| AL355574.1 | 0.222807 | 0.15234 | 0.325871 | 9.92E-15 |
| AL355974.2 | 1.64174 | 1.452644 | 1.855452 | 2.02E-15 |
| LINC02381 | 1.524586 | 1.309156 | 1.775466 | 5.77E-08 |
| LINC00632 | 0.362908 | 0.260521 | 0.505535 | 2.05E-09 |
| AL391807.1 | 0.650384 | 0.536438 | 0.788534 | 1.20E-05 |
| AL157700.1 | 0.504364 | 0.41581 | 0.611777 | 3.70E-12 |
| AL157392.3 | 0.106547 | 0.062314 | 0.182177 | 2.80E-16 |
| AC010624.2 | 0.434142 | 0.282999 | 0.666009 | 0.000133 |
| GAS5 | 0.619778 | 0.489484 | 0.784754 | 7.10E-05 |
| DICER1-AS1 | 0.285808 | 0.197863 | 0.412844 | 2.47E-11 |
| MIR4435-2HG | 2.102551 | 1.742509 | 2.536986 | 8.85E-15 |
| AC021739.2 | 0.609197 | 0.520223 | 0.713389 | 7.63E-10 |
| AC080038.1 | 1.62847 | 1.41821 | 1.869903 | 4.73E-12 |
| AL121768.1 | 0.288012 | 0.117674 | 0.704918 | 0.006418 |
| LINC01023 | 1.770821 | 1.422531 | 2.204387 | 3.15E-07 |
| AC093673.1 | 1.890867 | 1.574737 | 2.27046 | 8.81E-12 |
| NORAD | 0.559009 | 0.390771 | 0.799678 | 0.001454 |
| SLC6A1-AS1 | 0.508573 | 0.362949 | 0.712625 | 8.55E-05 |
| EPB41L4A-AS1 | 0.451754 | 0.356574 | 0.57234 | 4.62E-11 |
| AL604028.1 | 1.962425 | 1.379677 | 2.791313 | 0.000177 |
| AC013391.3 | 0.686326 | 0.59006 | 0.798298 | 1.05E-06 |
| AC026471.4 | 0.62512 | 0.458361 | 0.852549 | 0.003001 |
| AL365205.3 | 0.609465 | 0.453676 | 0.81875 | 0.00101 |
| AC103974.1 | 0.454874 | 0.347491 | 0.595441 | 9.84E-09 |
| AC104985.1 | 0.60292 | 0.437719 | 0.83047 | 0.001955 |
| AC008124.1 | 0.373057 | 0.251744 | 0.55283 | 8.95E-07 |
| LINC02283 | 0.738284 | 0.679235 | 0.802467 | 9.75E-13 |
| AL118505.1 | 0.630756 | 0.563626 | 0.705883 | 1.00E-15 |
| LINC00689 | 0.866056 | 0.783143 | 0.957746 | 0.005098 |
| AL512625.2 | 0.594502 | 0.480598 | 0.735403 | 1.65E-06 |
| TPT1-AS1 | 0.326255 | 0.199445 | 0.533693 | 8.17E-06 |
| AC012645.1 | 0.428015 | 0.29886 | 0.612986 | 3.65E-06 |
| AC053503.1 | 0.689903 | 0.620598 | 0.766948 | 6.32E-12 |
| NDUFA6-DT | 0.095838 | 0.053614 | 0.171316 | 2.51E-15 |
| AC105206.2 | 0.428529 | 0.300413 | 0.611283 | 2.93E-06 |
| AL133304.3 | 0.67872 | 0.556371 | 0.827974 | 0.000133 |
| AC021739.3 | 0.544432 | 0.440374 | 0.673078 | 1.93E-08 |
| AC254562.3 | 0.336639 | 0.255664 | 0.443261 | 8.80E-15 |
| LINC00609 | 0.665834 | 0.540362 | 0.820442 | 0.000135 |
| AC126407.1 | 0.612433 | 0.502041 | 0.747098 | 1.33E-06 |
| HCG18 | 0.449658 | 0.263237 | 0.768098 | 0.003436 |
| AC127502.2 | 3.368807 | 2.36866 | 4.791258 | 1.40E-11 |
| AC009113.1 | 0.356375 | 0.244383 | 0.519689 | 8.30E-08 |
| AC092720.2 | 0.558055 | 0.410341 | 0.758943 | 0.000201 |
| NNT-AS1 | 0.511477 | 0.349382 | 0.748773 | 0.000565 |
| TGFB2-AS1 | 1.547985 | 1.371162 | 1.747611 | 1.66E-12 |
| FAM13A-AS1 | 0.385134 | 0.204015 | 0.727044 | 0.003248 |
| AC015922.2 | 1.730929 | 1.434692 | 2.088332 | 1.01E-08 |
| AC122707.1 | 0.731382 | 0.619481 | 0.863498 | 0.000222 |
| BX293535.1 | 0.624038 | 0.48465 | 0.803514 | 0.000256 |
| AL645608.6 | 0.529078 | 0.434253 | 0.644609 | 2.66E-10 |
| NEAT1 | 1.506733 | 1.283484 | 1.768814 | 5.44E-07 |
| AC062021.1 | 0.696341 | 0.630587 | 0.768952 | 8.59E-13 |
| TRHDE-AS1 | 0.365034 | 0.194506 | 0.685068 | 0.001704 |
| AL590666.2 | 0.738984 | 0.64488 | 0.846821 | 1.35E-05 |
| LINC02609 | 1.647654 | 1.296677 | 2.093631 | 4.39E-05 |
| RPARP-AS1 | 0.167722 | 0.106145 | 0.26502 | 2.03E-14 |
| Z97989.1 | 0.553525 | 0.46218 | 0.662922 | 1.30E-10 |
| SNAI3-AS1 | 0.22699 | 0.133449 | 0.386101 | 4.47E-08 |
| LINC00237 | 0.642351 | 0.53924 | 0.765179 | 7.12E-07 |
| BCRP3 | 0.262447 | 0.146675 | 0.469598 | 6.60E-06 |
| AL162511.1 | 0.771775 | 0.664351 | 0.896569 | 0.000705 |
| DGCR5 | 0.529116 | 0.415483 | 0.673827 | 2.46E-07 |
| LINC02593 | 0.490938 | 0.400051 | 0.602474 | 9.69E-12 |
| AL021368.3 | 0.56518 | 0.420674 | 0.759325 | 0.000152 |
| CYTOR | 2.461899 | 2.01862 | 3.002519 | 5.85E-19 |
| AL354892.2 | 0.445421 | 0.345971 | 0.573458 | 3.53E-10 |
| LINC01089 | 0.668956 | 0.523525 | 0.854788 | 0.001307 |
| MIR9-3HG | 0.547118 | 0.481055 | 0.622254 | 4.09E-20 |
| MIR124-2HG | 0.653362 | 0.52964 | 0.805984 | 7.08E-05 |
| ZFHX2-AS1 | 0.128676 | 0.072598 | 0.228071 | 2.19E-12 |
| TTC28-AS1 | 0.346405 | 0.220458 | 0.544303 | 4.26E-06 |
| SLC9A3-AS1 | 0.686943 | 0.540132 | 0.873658 | 0.002206 |
| AC009041.2 | 0.676076 | 0.604617 | 0.75598 | 6.51E-12 |
| AF131215.5 | 0.599161 | 0.460982 | 0.778759 | 0.000128 |
